# Mutation Screening of Retinal Dystrophy Patients by Targeted Capture from Tagged Pooled DNAs and Next Generation Sequencing

**DOI:** 10.1371/journal.pone.0104281

**Published:** 2014-08-18

**Authors:** Christopher M. Watson, Mohammed El-Asrag, David A. Parry, Joanne E. Morgan, Clare V. Logan, Ian M. Carr, Eamonn Sheridan, Ruth Charlton, Colin A. Johnson, Graham Taylor, Carmel Toomes, Martin McKibbin, Chris F. Inglehearn, Manir Ali

**Affiliations:** 1 Yorkshire Regional Genetics Service, St. James's University Hospital, Leeds, United Kingdom; 2 Section of Ophthalmology & Neuroscience, Leeds Institute of Biomedical & Clinical Sciences, University of Leeds, Leeds, United Kingdom; 3 Section of Genetics, Leeds Institute of Biomedical & Clinical Sciences, University of Leeds, Leeds, United Kingdom; 4 Department of Ophthalmology, St. James's University Hospital, Leeds, United Kingdom; Radboud University Nijmegen Medical Centre, Netherlands

## Abstract

**Purpose:**

Retinal dystrophies are genetically heterogeneous, resulting from mutations in over 200 genes. Prior to the development of massively parallel sequencing, comprehensive genetic screening was unobtainable for most patients. Identifying the causative genetic mutation facilitates genetic counselling, carrier testing and prenatal/pre-implantation diagnosis, and often leads to a clearer prognosis. In addition, in a proportion of cases, when the mutation is known treatment can be optimised and patients are eligible for enrolment into clinical trials for gene-specific therapies.

**Methods:**

Patient genomic DNA was sheared, tagged and pooled in batches of four samples, prior to targeted capture and next generation sequencing. The enrichment reagent was designed against genes listed on the RetNet database (July 2010). Sequence data were aligned to the human genome and variants were filtered to identify potential pathogenic mutations. These were confirmed by Sanger sequencing.

**Results:**

Molecular analysis of 20 DNAs from retinal dystrophy patients identified likely pathogenic mutations in 12 cases, many of them known and/or confirmed by segregation. These included previously described mutations in *ABCA4* (c.6088C>T,p.R2030*; c.5882G>A,p.G1961E), *BBS2* (c.1895G>C,p.R632P), *GUCY2D* (c.2512C>T,p.R838C), *PROM1* (c.1117C>T,p.R373C), *RDH12* (c.601T>C,p.C201R; c.506G>A,p.R169Q), *RPGRIP1* (c.3565C>T,p.R1189*) and *SPATA7* (c.253C>T,p.R85*) and new mutations in *ABCA4* (c.3328+1G>C), *CRB1* (c.2832_2842+23del), *RP2* (c.884-1G>T) and *USH2A* (c.12874A>G,p.N4292D).

**Conclusions:**

Tagging and pooling DNA prior to targeted capture of known retinal dystrophy genes identified mutations in 60% of cases. This relatively high success rate may reflect enrichment for consanguineous cases in the local Yorkshire population, and the use of multiplex families. Nevertheless this is a promising high throughput approach to retinal dystrophy diagnostics.

## Introduction

Retinal dystrophies are to date the most genetically heterogeneous set of inherited conditions known to affect a single organ. This complicates genetic screening for conditions such as retinitis pigmentosa (RP), cone-rod dystrophy (CRD) and Leber congenital Amaurosis (LCA) since each can result from mutations in many genes (see RetNet, https://sph.uth.tmc.edu/retnet/) which, with the exception of LCA, follow dominant, recessive or X-linked patterns of inheritance. Nationally, inherited retinal disease accounts for 4.2% of all sight impairment certifications and 5.5% of blindness cases [1]. These diseases are a more significant issue in the West Yorkshire population due to the high incidence of first cousin marriage and consequent recessive disease in the local Pakistani community [2]. Until recently, patients could at best be offered only limited counselling based on approximate recurrence rates for a given mode of inheritance, whilst presymptomatic diagnosis and carrier status testing were impossible in all but a minority of cases. A further incentive for seeking to improve this situation is the notable success of an increasing number of clinical trials for gene and other targeted therapies for retinal dystrophies [3]–[7]. These are gene-specific, meaning that only patients for whom mutations have been identified will benefit from these novel approaches to stratified medicine.

In order to increase patient recruitment to new gene- or mutation-specific trials, several groups have already highlighted the potential of next generation sequencing in disease diagnosis [8]–[14]. Here we confirm the efficacy of this approach in a Northern UK cohort. In addition we describe the use of a previously published approach, tagging and DNA pooling prior to targeted capture and next generation sequencing [15], providing a valuable refinement to existing high throughput next generation sequencing strategies for identifying the genetic basis of retinal dystrophy.

## Materials and Methods

### Ethics Statement

Patients and their relatives recruited to the study gave informed, written consent using a process approved by the Leeds East Research Ethics committee (Project number 03/362), adhering to the tenets of the Declaration of Helsinki.

### Samples

**Figure 1:**
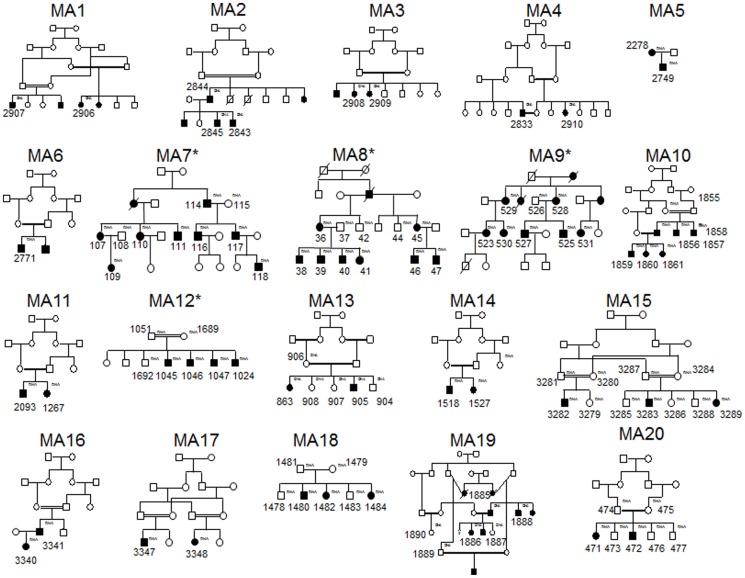
Family pedigrees of patients that were studied. Individuals from whom DNA was available are assigned the DNA notation in small lettering to the top right hand side of the symbol (and are also numbered). * highlights pedigrees that have been abbreviated for this figure.

The families were selected on the basis that there were multiple affected members with an unidentified molecular genetic diagnosis. The patients were diagnosed with a retinal dystrophy by an experienced ophthalmologist. Pedigree structures are depicted in Figure 1, while diagnoses, possible inheritance patterns, ethnicity and summary information regarding numbers of affected cases and members who were available for sampling are recorded in Table S1 in File S1. Peripheral blood was collected from affected patients, their parents and unaffected relatives where available. Genomic DNA was extracted from blood according to standard procedures.

### Target design

In order to enrich specific regions of the patient's genomic DNA, a liquid-phase reagent comprising ‘SureSelect Target Enrichment’ biotinylated cRNA baits was designed using the Agilent Technologies eArray software (http://www.genomics.agilent.com/) (Agilent Technologies UK Limited, Wokingham, UK). In total, 2,988 coding exons as well as a single intronic region, and their 100 bp flanking sequences, were selected in the UCSC genome database (http://www.genome.ucsc.edu/) from all of the 162 genes implicated in retinal degeneration (RetNet, July 2010). The list of genes targeted is shown in Table S2 in File S1. This consisted of 46,287 RNA baits at 5× tiling to cover 776.5 kb of DNA sequence. Probes could not be designed against 9 exons (Table S3 in File S1).

### Library construction and massively parallel sequencing

Genomic DNA was sheared using a Covaris S220 sonicator. Illumina sequencing adapters containing 6 bp sequence tags were ligated to the samples, with each DNA sample being ligated to a different tag. The tagged DNA libraries were pooled into batches and captured using the SureSelect custom baits according to the manufacturer's instructions. Each captured pool was sequenced using single-end 80 bp reads on an Illumina GAIIx Sequencer (Illumina Inc., Little Chesterford, UK) according to the manufacturer's instructions.

### Alignments and variant detection

Sequence data were generated in qseq format and barcode sorted by their unique 5′ tag using NovoSort. The sorted fastq files have been deposited in the European Nucleotide Archive (http://www.ebi.ac.uk/ena/) with study accession number, PRJEB6380. The reads were aligned to the human genome sequence, hg19, using Novoalign (v2.08.01). Following realignment around indels, the GATK (v2.0.34) Unified Genotyper was used to identify variants [16]. The output VCF files were annotated for analysis using Alamut-HT (v1.0.4) (Interactive Biosoftware, Rouen, France). Analysis of read depth was performed using BEDTools (v2.15.0) and the GATK Count Reads walker.

Variants were filtered to exclude those more than 5 bp beyond the splice site junction. Synonymous variants and those with minor allele frequencies ≥0.01 in dbSNP or the 1,000 genomes project were also excluded.

**Figure 2:**
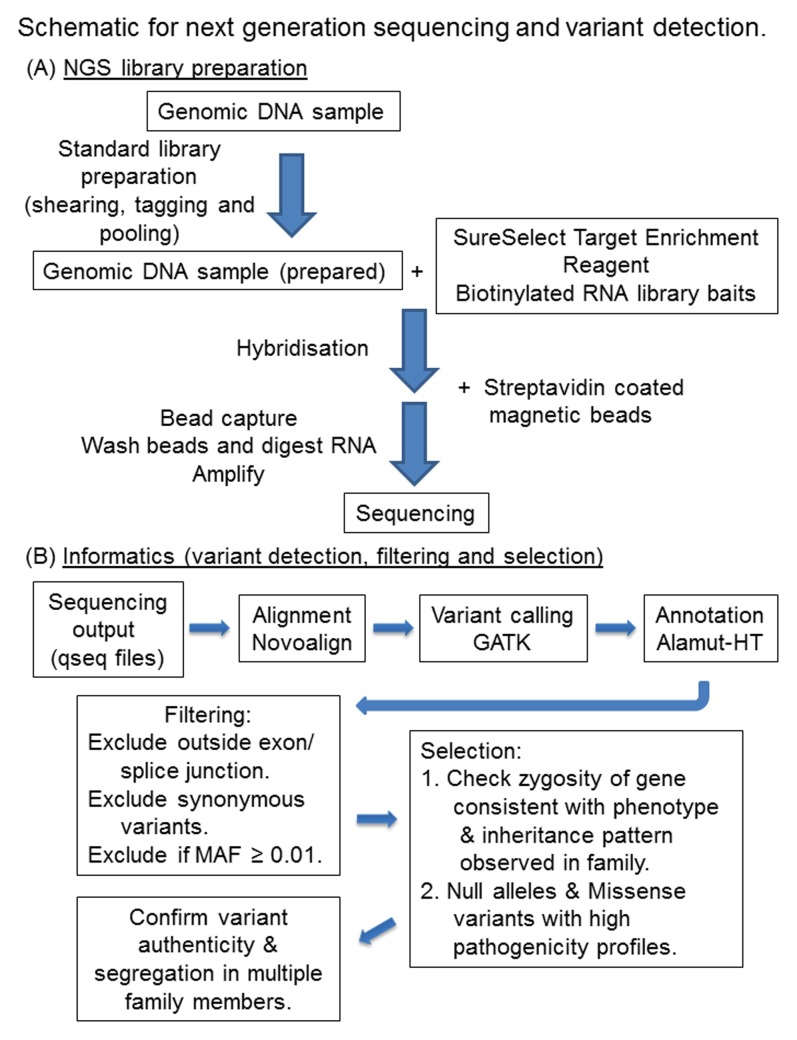
Schematic for next generation sequencing and variant detection. The strategy for NGS library preparation (A) and informatics used (B) are depicted.

From the remaining list, variants were then selected for further analysis if they met one or both of the following criteria. Firstly, variants that occurred in genes that had previously been associated with the observed phenotype and showed the expected pattern of inheritance were selected. Secondly, null alleles resulting from nucleotide deletions or insertions, premature stop codon mutations or changes affecting the conserved 2 bp adjacent to the splice site junction as well as missense variants with at least 2 out of 4 high pathogenicity scores were selected. For a high pathogenicity profile, scores recorded in the Alamut-HT report included BLOSUM62 (Blocks Substitution Matrix; http://www.uky.edu/Classes/BIO/520/BIO520WWW/blosum62.htm) <0, AGVGD (Align Grantham Variation and Grantham Deviation; http://agvgd.iarc.fr/agvgd_input.php) between C15 and C65, SIFT (Sorts Intolerant From Tolerant substitutions, http://sift.jcvi.org) <0.05 or deleterious and MAPP (Multivariate Analysis of Protein Polymorphism; http://mendel.stanford.edu/SidowLab/downloads/MAPP) = bad. A schematic for the sequencing and informatics pipeline is shown in Figure 2. For any cases with a diagnosis of LCA, the unfiltered variant lists were also analysed for the deep intronic mutation c.2991+1655A>G in *CEP290* that causes this phenotype [17].

### Sanger sequencing of potential disease-causing variants

Variants selected by the above criteria were confirmed by conventional Sanger sequencing of patient genomic DNA using the BigDye terminator cycle sequencing kit (Applied Biosystems, Paisley, UK) on an ABI3130xl sequencer (Applied Biosystems) and analysed using Sequencing Analysis v.5.2 software (Applied Biosystems). This was used to confirm presence of the mutation and test whether the mutation segregated with the disease phenotype in the family in question.

Confirmed pathogenic mutations were deposited in the publicly available LOVD database (http://databases.lovd.nl/shared/).

## Results

### Validating the capture reagent and establishing a pipeline for variant detection

To test the feasibility of identifying pathogenic mutations in genomic DNA from patients with retinal degeneration, we selected four patients in whom, by Sanger sequencing of candidate genes, we had identified mutation(s) deemed clearly causative based on exclusion from control cohorts, predicted pathogenicity and segregation in additional family members. The analysis of the data for this study was conducted by one of the co-authors (David A Parry) without prior knowledge of these known mutations in the samples. Briefly, a sequencing adapter containing a different 6 bp sequence tag was ligated to each patient's sonicated DNA. The tagged aliquots were pooled prior to hybridisation against the target enrichment reagent and run on a single lane of the Illumina GAIIx DNA sequencer. The sequence data for each sample was sorted by sequence tag and aligned against the human reference sequence for analysis of coverage and read depth (Table 1). Pooling of 4 samples gave a range of coverage between 95.6% to 96.9% with at least 20 good quality reads following duplicate removal and between 1 and 2% that had less than 5× read depth. A list of variants was generated for each sample and these were filtered without family history information according to the criteria highlighted in Table 2, and described in the Methods section, to give rise to a list of candidate variants for each sample (Table S4 in File S1).

**Table 1:** Coverage and read depth following targeted capture and next generation sequencing for the 4 patient verification study.

Sample	Tag	Aligned Reads	Reads on target	% Reads on target	Mean coverage	% ≥5	% ≥10	% ≥15	% ≥20*	% ≥30	% ≥50
Patient A tag1	CAACCT	6,296,720	1,310,107	20.8%	133	98.5	97.7	96.8	95.7	93.3	86.5
Patient B tag2	AACCAT	5,788,340	1,359,182	23.5%	137	98.4	97.5	96.6	95.6	93.4	87.9
Patient C tag3	AAGGAT	8,539,613	2,174,816	25.5%	220	98.7	98.2	97.6	96.9	95.9	93.1
Patient D tag4	AATTAT	4,314,207	1,609,443	37.3%	164	98.5	97.6	96.7	95.8	93.9	88.8
Patient tag1 to 4, AVERAGE						98.5	97.8	96.9	96.0	94.1	89.1

The tagging, aligned reads, reads on target, % reads on target, mean coverage and % coverage with a particular minimum read depth are shown for each patient DNA. The asterix highlights the % coverage with a greater than or equal to 20 read depth.

**Table 2:** Filtering the variant lists following targeted capture and next generation sequencing for the 4 patient verification study.

Filtering process	Patient A	Patient B	Patient C	Patient D
Total variants identified	614	564	595	580
Exclude outside exon/splice junction	278	282	269	260
Exclude synonymous variants	134	142	131	124
Exclude if MAF ≥0.01	7	12	10	3

Exon constitutes coding variants only. Splice junction constitutes +/−5 bp around an exon. A full list of variants is shown in Table S4.

Prioritisation of the variants was based on whether the genotype was consistent with disease symptoms in the family, the variant type and pathogenicity scores. For sample A with a diagnosis of RP, heterozygous mutations in *RP9*, *RP1* and *FSCN2* were deemed consistent with disease symptoms, and of these a high pathogenicity profile suggested that the strongest candidate for causation in sample A was the *RP9* variant. For sample B, though a number of changes were observed, only compound heterozygosity for a premature stop codon and a high pathogenicity missense mutation in *CRB1* fitted with the LCA diagnosis in this patient. For sample C, heterozygous variants in *RP1* and a homozygous variant in *USH2A* were considered possible candidates for causing RP in this patient. However based on pathogenicity scores and variant type, the strongest candidates for disease causation in sample C were the *RP1* variants. For sample D, only a null mutation in *PRPF31* was identified as consistent with the diagnosis of RP.

The variants that had previously been deemed causative in each sample are shown in Table 3. As these variants had indeed been implicated as candidates for pathogenicity following filtering and prioritisation as highlighted above, without the need for segregation analysis, this confirmed that the pipeline used to identify pathogenic mutations was robust.

**Table 3:** The previously identified pathogenic mutations in the 4 patient study.

Patient	Diagnosis	Inheritance Pattern	Chr	Position	Gene	Coding Effect	cDNA change	Protein change	BLOSUM62	AGVGDclass	SIFTprediction	MAPPprediction	Zygosity
A	RP	Dom.	7	33136162	*RP9*	missense	NM_203288.1:c.410A>T	p.His137Leu	***−3***	***C15***	***Deleterious***	***bad***	Het
B	LCA	Rec.	1	197390534	*CRB1*	nonsense	NM_201253.2:c.1576C>T	p.Arg526*	NA	NA	NA	NA	Het
			1	197404300	*CRB1*	missense	NM_201253.2:c.3307G>A	p.Gly1103Arg	***−2***	***C25***	***Deleterious***	***bad***	Het
C	RP	Dom.	8	55538727	*RP1*	frameshift	NM_006269.1:c.2285_2289delTAAAT	p.Leu762Tyrfs*17	NA	NA	NA	NA	Het
D	RP	Dom.	19	54621976	*PRPF31*	frameshift	NM_015629.3:c.201delT	p.Ile67Metfs*14	NA	NA	NA	NA	Het

The chromosome and position of the mutation is depicted according to the human genome assembly, hg19. Text in bold and italicised highlights high pathogenicity of missense variants. For BLOSUM62, high pathogenicity = <0; AGVGD, high pathogenicity = C15 to C65; SIFT prediction, high pathogenicity = deleterious; MAPP prediction, high pathogenicity = bad. NA = not annotated.

### Screening patients with unknown mutations

We then selected 20 patients with various retinal degenerations for which no mutation had yet been identified and performed the pre-capture pooling procedure on the tagged DNA libraries pooled in batches of four samples. Following alignment, variant detection and filtering as described in the Methods, a list of candidate variants were identified for each sample (Table S5 in File S1). Candidate variants were prioritised as described previously and Sanger sequenced to confirm the presence of the mutation. Segregation was performed where DNA from other family members was available.

For MA1, family history suggested LCA with recessive inheritance caused by an autozygous mutation. The variant list following analysis of patient 2906 (a female) suggested the homozygous *CRB1* mutation (c.2832_2842+23del) as the only candidate consistent with the diagnosis in the family [18]. Analysis of the other affected case from whom DNA was available (2907) confirmed the *CRB1* mutation as the pathogenic cause of disease.

For MA2, family history of the index case (2844, a male) with unaffected parents and consanguinity suggested recessive inheritance caused by an autozygous mutation. The variant list following analysis of this case suggested a previously-identified homozygous nonsense mutation in *ABCA4* (c.6088C>T, p.R2030*) [19] consistent with a diagnosis of CRD as the primary candidate. This mutation was indeed confirmed in the index case and subsequently found to be heterozygous in his affected offspring (2843 and 2845) suggesting that they both had an unidentified *ABCA4* mutation on their other allele which they had inherited from their mother.

For MA3, family history suggested RP with recessive inheritance due to an autozygous mutation. The variant list following analysis of patient 2908 (a female) identified a homozygous missense variant in *USH2A* (c.12874A>G, p.N4292D) with a high pathogenicity profile as the sole candidate. The *USH2A* mutation was indeed subsequently confirmed in both affected cases from whom DNA was available.

For MA4, family history suggested recessive inheritance of RP and an autozygous mutation. The variant list following analysis of case 2833 (a male) highlighted two homozygous missense variants in *EYS* as possible candidates. Following analysis of the other affected case (2910), both *EYS* variants were homozygous and Sanger sequencing of the *EYS* terminal exon that was not covered by the capture reagent failed to identify any other changes. One of the *EYS* variants (c.7558T>C, p.F2520L) disrupts the second laminin G subdomain which is essential for normal protein function [20]. Given the degree of co-segregation and consistency with phenotype, this was considered the most likely variant to be pathogenic, but given the low pathogenicity profile scores due to the lack of amino acid conservation of the normal residue in vertebrates (data not shown), the variant was considered unproven.

For MA5 family history suggests dominant inheritance of a CRD phenotype. The variant list following analysis of patient 2278 (a female) did not highlight any obvious candidates.

For MA6, family history suggested recessive inheritance of RP with an autozygous mutation. The variant list described a previously identified homozygous missense mutation in *RDH12* (c.601T>C, p.C201R) [21] with a high pathogenicity profile which was confirmed in the case (a male) as the likely cause of disease.

For MA7, family history suggested dominant inheritance of CRD. The variant list following analysis of patient 114 (a male) highlighted the heterozygous *PROM1* mutation (c.1117C>T, p.R373C) which was previously identified in patients with a diagnosis of cone-rod dystrophy [22], [23] as the possible cause of disease symptoms. This was confirmed by segregation in the family.

For MA8, family history suggested dominant or X-linked inheritance of RP with macular involvement. The variant list derived from analysing case 40 (a male) described a dominant variant in *NR2E3* and an X-linked variant in *RP2* as the most likely candidates. Analysis of the variants in additional family members for segregation identified that only the splicing variant in *RP2* (c.884-1G>T) followed disease symptoms as X-linked dominant inheritance in the family.

For MA9, family history suggested dominant inheritance of a macular dystrophy phenotype. The variant list derived from analysing case 530 (a female) identified heterozygous variants in *HMCN1* and the previously reported *GUCY2D* [24], [25] as the most likely candidates. Analysis of additional family members from whom DNA was available only confirmed segregation of the *GUCY2D* mutation (c.2512C>T, p.R838C) with disease symptoms in the family.

For MA10, family history suggested recessive inheritance of CRD with an autozygous mutation. The variant list from analysing case 1857 (a male) highlighted only one candidate, a homozygous null variant in *RPGRIP1* (c.3565C>T, p.R1189*) that was recently reported independently as a pathogenic cause of disease [26]. Segregation analysis confirmed this mutation as the cause of disease symptoms in this family.

For MA11, family history suggested recessive RP with an autozygous mutation. The variant list derived from analysing patient 2093 (a male) described a homozygous missense variant in *BBS2* (c.1895G>C, p.R632P) as the most likely candidate. Analysis of the other affected case 1267 confirmed that the *BBS2* mutation, which was recently reported to be a common cause of RP in the Ashkenazi Jewish population [27], was the likely pathogenic cause of disease.

For MA12, family history suggested recessive CRD. The variant list derived from case 1024 (a male) highlighted two heterozygous missense variants in *CDH23* as possible candidates even though recessive mutations in this gene usually cause Usher syndrome. The absence of segregation in other family members suggested that these variants were not the pathogenic cause of disease in this family.

For MA13, family history suggested recessive inheritance of RP. Analysis of the variant list from case 863 (a female) identified missense variants in *GPR98* and *MYO7A* as the best candidates even though mutations in these genes usually cause recessive Usher syndrome. On the basis of higher pathogenicity profiles, the *GPR98* variants were analysed further. Segregation analysis confirmed that these variants were not the cause of disease symptoms in this family.

For MA14, family history suggested RP with recessive inheritance due to an autozygous mutation in each case. The variant lists for patient 1518 (a male), identified two heterozygous variants in *BBS12* and one in *FSCN2* as possible candidates though neither option appeared to fit the observed phenotype perfectly. Following analysis of the other affected sibling (1527) these variants did not segregate with the disease phenotype and so were unlikely to be the pathogenic cause of disease in this family.

For MA15, family history suggested recessive CRD with an autozygous mutation. The variant list for patient 3283 (a male) identified a previously been reported homozygous null variant in *SPATA7* (c.253C>T, p.R85*) [28] as the most likely candidate. Analysis of DNA from other family members highlighted that this variant segregated with the disease phenotype as expected.

For MA16 with a diagnosis of LCA, family history of the index case (3341, a male) suggested recessive inheritance and an autozygous mutation. The variant list from analysing 3340 highlighted only the previously reported LCA causing *RDH12* variant (c.506G>A, p.R169Q) [29] as the likely cause of disease. This mutation was confirmed in the other family member.

For MA17, family history suggested recessive inheritance of RCD caused by an autozygous mutation. From the variant list of patient 3347 (a male), no obvious candidates could be identified.

For MA18, family history suggested CRD with recessive inheritance. From analysing the variant list of case 1484 (a female), compound heterozygous variants in *ABCA4* for the previously reported missense variant (c.5882G>A, p.G1961E) [30], [31] as well as the heterozygous splicing variant (c.3328+1G>C) suggested these changes as the most likely to account for the CRD in this family. This was confirmed by segregation analysis of the variants.

For MA19 family history suggested recessive inheritance of RCD with recessive inheritance with an autozygous mutation. The variant list of patient 1885 (a male), identified compound heterozygous variants in *CC2D2A* and *PCDH15* as well as a variant in *WFS1* with a high pathogenicity profile as possible candidates though none of the options appeared to fit the observed phenotype perfectly. Analysis of family members from whom DNA was available confirmed three of the putative variants were artefacts and the remaining ones in *CC2D2A* and *WFS1* did not segregate with disease.

For MA20, family history suggested RP with recessive inheritance due to an autozygous mutation. The variant list of case 472 (a male) identified a single homozygous missense variant in *TRPM1* as well as compound heterozygous variants in *CEP290* and a variant in *CA4*, though none of these candidates appeared to exactly fit the observed phenotype. As suspected, these variants were either artefacts or failed to segregate with disease in this family suggesting that the pathogenic cause of disease has yet to be identified.

Using this approach likely pathogenic mutation(s) were identified in 12 out of 20 cases (60%). A list of these mutations is highlighted in Table 4 and the sequence chromatograms of each candidate variant highlighted in Figure S1 in File S1. To summarise, the mutations consisted of previously reported mutations of clinical significance in *ABCA4* (c.6088C>T, p.R2030* [19] and c.5882G>A, p.G1961E [30], [31]), *RDH12* (c.601T>C, p.C201R [21] and c.506G>A, p.R169Q [29]), *PROM1* (c.1117C>T, p.R373C [22], [23]), *GUCY2D* (c.2512C>T, p.R838C [24], [25]), *RPGRIP1* (c.3565C>T, p.R1189* [26]), *BBS2* (c.1895G>C, p.R632P [27]) and *SPATA7* (c.253C>T, p.R85* [28]) and new mutations in *CRB1* (c.2832_2842+23del), *USH2A* (c.12874A>G, p.N4292D), *RP2* (c.884-1G>T) and *ABCA4* (c.3328+1G>C). Of the 8 cases for which the pathogenic mutation could not be identified, the absence of zero-coverage targeted regions suggested that a homozygous deletion removing an exon(s) was not the cause of disease in these patients.

**Table 4:** List of confirmed likely pathogenic mutations in the 20 patient study.

ID	Diagnosis	Inheritance Pattern	Chr	Position	Gene	Coding Effect	cDNA change	Protein change	BLOSUM62	AGVGD class	SIFT prediction	MAPP prediction	Zygosity	[Reference]
MA1	LCA	Rec.	1	197398744	*CRB1*	frameshift	NM_201253.2:c.2832_2842+23del	p.?	NA	NA	NA	NA	Homo	
MA2	CRD	Rec./Dom.	1	94471056	*ABCA4*	nonsense	NM_000350.2:c.6088C>T	p.Arg2030*	NA	NA	NA	NA	Homo	[19]
MA3	RP	Rec.	1	215848379	*USH2A*	missense	NM_206933.2:c.12874A>G	p.Asn4292Asp	1	***C15***	***Deleterious***	***bad***	Homo	
MA4	RP	Rec.	None confirmed											
MA5	CRD	Dom.	None confirmed											
MA6	RP	Rec.	14	68193850	*RDH12*	missense	NM_152443.2:c.601T>C	p.Cys201Arg	***−3***	C0	***Deleterious***	***bad***	Homo	[21]
MA7	CRD	Dom.	4	16014922	*PROM1*	missense	NM_006017.2:c.1117C>T	p.Arg373Cys	***−3***	C0	***Deleterious***	***bad***	Het	[22], [23]
MA8	RP with maculopathy	Dom./X-link.	X	46736939	*RP2*	splicing	NM_006915.2:c.884-1G>T	p.?	NA	NA	NA	NA	Homo	
MA9	MD	Dom.	17	7918018	*GUCY2D*	missense	NM_000180.3:c.2512C>T	p.Arg838Cys	***−3***	***C65***	***Deleterious***	***bad***	Het	[24], [25]
MA10	CRD	Rec.	14	21813304	*RPGRIP1*	nonsense	NM_020366.3:c.3565C>T	p.Arg1189*	NA	NA	NA	NA	Homo	[26]
MA11	RP	Rec.	16	56530894	*BBS2*	missense	NM_031885.3:c.1895G>C	p.Arg632Pro	***−2***	***C15***	Tolerated	***bad***	Homo	[27]
MA12	CRD	Rec.	None confirmed											
MA13	RP	Rec.	None confirmed											
MA14	RP	Rec.	None confirmed											
MA15	CRD	Rec.	14	88883069	*SPATA7*	nonsense	NM_018418.4:c.253C>T	p.Arg85*	NA	NA	NA	NA	Homo	[28]
MA16	LCA	Rec.	14	68193755	*RDH12*	missense	NM_152443.2:c.506G>A	p.Arg169Gln	1	***C35***	***Deleterious***	***bad***	Homo	[29]
MA17	RCD	Rec.	None confirmed											
MA18	CRD	Rec.	1	94508316	*ABCA4*	splicing	NM_000350.2:c.3328+1G>C	p.?	NA	NA	NA	NA	Het	
				94473807	*ABCA4*	missense	NM_000350.2:c.5882G>A	p.Gly1961Glu	***−2***	***C65***	***Deleterious***	***bad***	Het	[30], [31]
MA19	RCD	Rec.	None confirmed											
MA20	RP	Rec.	None confirmed											

The ID and diagnosis of the cases studied as well as the chromosome and position of the mutation according to the human genome assembly hg19, gene, coding effect, cDNA and protein nomenclature, BLOSUM62, AGVGD class, SIFT prediction, MAPP prediction, zygosity and whether the mutation has been previously implicated into causing disease are shown. Text in bold and italicised highlights high pathogenicity prediction for missense variants. For BLOSUM62, high pathogenicity = <0; AGVGD, high pathogenicity = C15 to C65; SIFT prediction, high pathogenicity = deleterious; MAPP prediction, high pathogenicity = bad. NA = not annotated. Homo = homozygous. Het = heterozygous.

## Discussion

In this paper we describe a previously published strategy for target capture and next generation sequencing that utilises tagging and pooling of DNAs in batches of four prior to enrichment [15]. This approach refines the use of targeted capture technology, facilitating the enrichment of exons from pooled samples using a single aliquot of capture reagent. This strategy differs from previously described methods which usually pool samples after the hybridization step to multiplex onto one lane of the sequencer. The technology described herein will contribute to the development of a retinal dystrophy diagnostic screening service by reducing costs associated with using a single capture reagent to analyse up to four samples in a single experiment. We also describe use of a reagent designed to enrich patient genomic DNA for all retinal dystrophy genes that were listed in Retnet as of July 2010. A recent update in January 2014 has 66 additional genes found to have mutations causing retinal dystrophy that were not included in the reagent used in this study. The flexibility of our approach means that these genes can be incorporated into subsequent versions of the targeted reagent. A methodological drawback of the targeted hybridisation approach is that regions containing repeat sequences cannot be adequately covered due to binding of the target DNA to multiple sites of repetitive sequence. In the current reagent, 9 exons including the *RPGR* ORF15 could not be covered because of repeat sequence, suggesting that these exons will have to be sequenced using alternative methods. In terms of data analysis, we observed a number of sequencing artefacts that may be due to low coverage, low sequence quality or the pooling of DNA samples but the most likely source was due to variant calling. In order to reduce the number of false negative results the stringency of variant calling algorithm was relaxed. This encompassing approach to capture all possible variants inevitably meant that there were also a number of false positives in the annotated variant lists.

The use of next generation sequencing for retinal disease diagnosis has been previously described (see Table 5). Researchers have used different target enrichment methods such as solid phase capture arrays [9], [12], [14] or PCR amplicons based approaches [8], [11] as opposed to liquid phase capture [10], [13] and have run the libraries on different machines such as the Roche 454 [8], [12], [14] or the ABI SOLiD [13] rather than the Illumina Genome Analyser [8]–[11]. Success in identifying the pathogenic mutation has, to date varied from 18% (3 out of 17 cases studied) [11] to 60% (3 out of 5 cases studied) [9] and there does not appear to be any correlation between successfully identifying the pathogenic mutation and the library preparation method or machine used for the study. The approach described in this paper gave a 60% (12 out of 20 cases studied) success rate, which is higher than the majority of previous studies. One possible reason for this may be that we focussed on studying families with multiple affected members rather than single cases with no family history. This allowed us to assess the pathogenicity of candidate disease causing variants by following the transmission of the mutation with the disease phenotype. It is interesting to note when studying isolated cases that several examples of *de novo* mutations as the cause of disease have been demonstrated [12], [14]. Another possible reason for the increased detection rate in this study is the high number of consanguineous cases in the local Yorkshire population, which allows filtering on the basis of homozygosity.

**Table 5:** Comparison of the methodological approaches in recent publications that have used high throughput next generation sequencing for retinal disease diagnosis.

Authors [Reference]	Detecting phenotypes	Library preparation		NGS instrument	Number of independent samples tested	Pathogenic mutation identified (%)
		Gene number	Method			
Bowne et al [8]	adRP	46	PCR amplicons	454GS FLX Titanium (Roche) & GAIIx (Illumina)	21	5 (24%)
Simpson et al [9]	RP	45	Solid phase customised capture array (NimbleGen)	GAIIx (Illumina)	5	3 (60%)
Coppieters et al [11]	LCA	16	PCR amplicons	GAIIx (Illumina)	17	3 (18%)
Neveling et al [12]	RP	111	Solid phase customised capture array (NimbleGen)	454GS FLX Titanium (Roche)	100	36 (36%)
Audo et al [10]	RD	254	Liquid phase targeted SureSelect capture (Agilent)	GAIIx (Illumina)	13	7 (54%)
O'Sullivan et al [13]	RD	105	Liquid phase targeted SureSelect capture (Agilent)	SOLiD 4 (Life Technologies)	50	21 (42%)
Shanks et al [14]	RP & CRD	73	Solid phase customised capture array (NimbleGen)	454GS FLX Titanium (Roche)	36	9 (25%)
Watson et al [This paper]	RD	162 (Retnet, July 2010)	Liquid phase targeted SureSelect capture (Agilent)	GAIIx (Illumina)	20	12 (60%)

adRP = autosomal dominant retinitis pigmentosa; CRD = cone rod dystrophy; LCA = leber congenital amaurosis;

RD = retinal dystrophies; RP = retinitis pigmentosa.

Patient feedback has highlighted the need for, and perceived value of, a definitive diagnosis based on genetic testing, and has shown that patients are motivated by a variety of factors to seek genetic testing [32]. Individuals may see many different eye specialists before a definitive diagnosis is made, whereas genetic testing can rapidly provide an accurate diagnosis. Furthermore, a genetic diagnosis can confirm the way in which the condition is inherited, giving clearer estimates of risk for patients and their relatives thus informing family planning decisions. Genetic testing can also facilitate pre-implantation diagnosis or prenatal testing as well as carrier testing in those who wish to know. In some cases such information may lead to improvements in therapy or direct patients towards trials for new potential therapies. It can also provide patients with an accurate guide to future function. Using this information, individuals can make informed decisions regarding education, employment and lifestyle.

To conclude, we report here that tagging DNA and pooling samples prior to hybridisation capture and next generation sequencing is a viable high throughput method for the genetic diagnosis of retinal dystrophies. This approach leaves a residual cohort of patients and families with retinal dystrophy that could not be resolved using the methods described. Their mutations may be in the known genes within regions that were not targeted such as the regulatory or intronic regions or one of the 9 exons of repetitive sequence. Alternatively, the mutation may be a cryptic splice site created by one of the synonymous variants that were removed during filtering. On the other hand, the mutation may be in one of the 66 additional genes that have been added to RetNet since the capturing reagent was manufactured, or it may be in a new gene that has never been implicated in retinal dystrophy. Nevertheless, this cohort serves as a powerful resource for further gene and mutation discovery by whole exome as well as genome sequencing.

## Supporting Information

File S1 **Supplementary figure and tables.**
(PDF)

## References

[1] Bunce C, Xing W, Wormald R (2010) Causes of blind and partial sight certifications in England and Wales: April 2007–March 2008. Eye 24: 1692–1699. 20847749 10.1038/eye.2010.122

[2] Darr A, Modell B (1988) The frequency of consanguineous marriage among British Pakistanis. J Med Genet 25: 186–190. 3351906 10.1136/jmg.25.3.186 PMC1015484

[3] Bainbridge JW, Smith AJ, Barker SS, Robbie S, Henderson R, et al (2008) Effect of gene therapy on visual function in Leber's congenital amaurosis. N Engl J Med 358: 2231–2239. 18441371 10.1056/NEJMoa0802268

[4] Maguire AM, Simonelli F, Pierce EA, Pugh EN Jr, Mingozzi F, et al (2008) Safety and efficacy of gene transfer for Leber's congenital amaurosis. N Engl J Med 358: 2240–2248. 18441370 10.1056/NEJMoa0802315 PMC2829748

[5] Lamba DA, Gust J, Reh TA (2009) Transplantation of human embryonic stem cell-derived photoreceptors restores some visual function in Crx-deficient mice. Cell Stem Cell 4: 73–79. 19128794 10.1016/j.stem.2008.10.015 PMC2713676

[6] Tan MH, Smith AJ, Pawlyk B, Xu X, Liu X, et al (2009) Gene therapy for retinitis pigmentosa and Leber congenital amaurosis caused by defects in AIPL1: effective rescue of mouse models of partial and complete Aipl1 deficiency using AAV2/2 and AAV2/8 vectors. Hum Mol Genet 18: 2099–2114. 19299492 10.1093/hmg/ddp133 PMC2722233

[7] Han Z, Conley SM, Makkia RS, Cooper MJ, Naash MI (2012) DNA nanoparticle-mediated ABCA4 delivery rescues Stargardt dystrophy in mice. J Clin Invest 122: 3221–3226. 22886305 10.1172/JCI64833 PMC3428101

[8] Bowne SJ, Sullivan LS, Koboldt DC, Ding L, Fulton R, et al (2011) Identification of disease-causing mutations in autosomal dominant retinitis pigmentosa (adRP) using next-generation DNA sequencing. Invest Ophthalmol Vis Sci 52: 494–503. 20861475 10.1167/iovs.10-6180 PMC3053293

[9] Simpson DA, Clark GR, Alexander S, Silvestri G, Willoughby CE (2011) Molecular diagnosis for heterogeneous genetic diseases with targeted high-throughput DNA sequencing applied to retinitis pigmentosa. J Med Genet 48: 145–151. 21147909 10.1136/jmg.2010.083568

[10] Audo I, Bujakowska KM, Léveillard T, Mohand-Said S, Lancelot ME, et al (2012) Development and application of a next-generation-sequencing (NGS) approach to detect known and novel gene defects underlying retinal diseases. Orphanet J Rare Dis 7: 8. 22277662 10.1186/1750-1172-7-8 PMC3352121

[11] Coppieters F, De Wilde B, Lefever S, De Meester E, De Rocker N, et al (2012) Massively parallel sequencing for early molecular diagnosis in leber congenital amaurosis. Genet Med 14: 576–585. 22261762 10.1038/gim.2011.51

[12] Neveling K, Collin RW, Gilissen C, van Huet RA, Visser L, et al (2012) Next-generation genetic testing for retinitis pigmentosa. Hum Mutat 33: 963–972. 22334370 10.1002/humu.22045 PMC3490376

[13] O'Sullivan J, Mullaney BG, Bhaskar SS, Dickerson JE, Hall G, et al (2012) A paradigm shift in the delivery of services for diagnosis of inherited retinal disease. J Med Genet 49: 322–326. 22581970 10.1136/jmedgenet-2012-100847

[14] Shanks ME, Downes SM, Copley RR, Lise S, Broxholme J, et al (2013) Next-generation sequencing (NGS) as a diagnostic tool for retinal degeneration reveals a much higher detection rate in early-onset disease. Eur J Hum Genet 21: 274–280. 22968130 10.1038/ejhg.2012.172 PMC3573204

[15] Harakalova M, Mokry M, Hrdlickova B, Renkens I, Blankensteijn JD, et al (2011) Multiplexed array-based and in-solution genomic enrichment for flexible and cost-effective targeted next-generation sequencing. Nat Protoc 6: 1870–1886. 22051800 10.1038/nprot.2011.396

[16] DePristo MA, Banks E, Poplin R, Garimella KV, Maguire JR, et al (2011) A framework for variation discovery and genotyping using next-generation DNA sequencing data. Nat Genet 43: 491–498. 21478889 10.1038/ng.806 PMC3083463

[17] den Hollander AI, Koenekoop RK, Yzer S, Lopez I, Arends ML, et al (2006) Mutations in the CEP290 (NPHP6) gene are a frequent cause of Leber congenital amaurosis. Am J Hum Genet 79: 556–561. 16909394 10.1086/507318 PMC1559533

[18] Lotery AJ, Jacobson SG, Fishman GA, Weleber RG, Fulton AB, et al (2001) Mutations in the CRB1 gene cause Leber congenital amaurosis. Arch Ophthalmol 119: 415–420. 11231775 10.1001/archopht.119.3.415

[19] Singh HP, Jalali S, Hejtmancik JF, Kannabiran C (2006) Homozygous null mutations in the ABCA4 gene in two families with autosomal recessive retinal dystrophy. Am J Ophthalmol 141: 906–913. 16546111 10.1016/j.ajo.2005.12.009

[20] Khan MI, Collin RW, Arimadyo K, Michael S, Azam M, et al (2010) Missense mutations at homologous positions in the fourth and fifth laminin A G-like domains of eyes shut homolog cause autosomal recessive retinitis pigmentosa. Mol Vis 16: 2753–2759. 21179430 PMC3003713

[21] Sun W, Gerth C, Maeda A, Lodowski DT, Van Der Kraak L, et al (2007) Novel RDH12 mutations associated with Leber congenital amaurosis and cone-rod dystrophy: biochemical and clinical evaluations. Vision Res 47: 2055–2066. 17512964 10.1016/j.visres.2007.04.005 PMC2441904

[22] Yang Z, Chen Y, Lillo C, Chien J, Yu Z, et al (2008) Mutant prominin 1 found in patients with macular degeneration disrupts photoreceptor disk morphogenesis in mice. J Clin Invest 118: 2908–2916. 18654668 10.1172/JCI35891 PMC2483685

[23] Michaelides M, Gaillard MC, Escher P, Tiab L, Bedell M, et al (2010) The PROM1 mutation p.R373C causes an autosomal dominant bull's eye maculopathy associated with rod, rod-cone, and macular dystrophy. Invest Ophthalmol Vis Sci 51: 4771–4780. 20393116 10.1167/iovs.09-4561 PMC2941169

[24] Van Ghelue M, Eriksen HL, Ponjavic V, Fagerheim T, Andreasson S, et al (2000) Autosomal dominant cone-rod dystrophy due to a missense mutation (R838C) in the guanylate cyclase 2D gene (GUCY2D) with preserved rod function in one branch of the family. Ophthalmic Genet 21: 197–209. 11135490

[25] Wilkie SE, Newbold RJ, Deery E, Walker CE, Stinton I, et al (2000) Functional characterization of missense mutations at codon 838 in retinal guanylate cyclase correlates with disease severity in patients with autosomal dominant cone-rod dystrophy. Hum Mol Genet 9: 3065–3073. 11115851 10.1093/hmg/9.20.3065

[26] Abu-Safieh L, Alrashed M, Anazi S, Alkuraya H, Khan AO, et al (2013) Autozygome-guided exome sequencing in retinal dystrophy patients reveals pathogenetic mutations and novel candidate disease genes. Genome Res 23: 236–247. 23105016 10.1101/gr.144105.112 PMC3561865

[27] Fedick A, Jalas C, Abeliovich D, Krakinovsky Y, Ekstein J, et al (2014) Carrier frequency of two BBS2 mutations in the Ashkenazi population. Clin Genet 85: 578–582. 23829372 10.1111/cge.12231

[28] Mackay DS, Ocaka LA, Borman AD, Sergouniotis PI, Henderson RH, et al (2011) Screening of SPATA7 in patients with Leber congenital amaurosis and severe childhood-onset retinal dystrophy reveals disease-causing mutations. Invest Ophthalmol Vis Sci 52: 3032–3038. 21310915 10.1167/iovs.10-7025

[29] Mackay DS, Dev Borman A, Moradi P, Henderson RH, Li Z, et al (2011) RDH12 retinopathy: novel mutations and phenotypic description. Mol Vis 17: 2706–2716. 22065924 PMC3209419

[30] Cella W, Greenstein VC, Zernant-Rajang J, Smith TR, Barile G, et al (2009) G1961E mutant allele in the Stargardt disease gene ABCA4 causes bull's eye maculopathy. Exp Eye Res 89: 16–24. 19217903 10.1016/j.exer.2009.02.001 PMC2742677

[31] Burke TR, Fishman GA, Zernant J, Schubert C, Tsang SH, et al (2012) Retinal phenotypes in patients homozygous for the G1961E mutation in the ABCA4 gene. Invest Ophthalmol Vis Sci 53: 4458–4467. 22661473 10.1167/iovs.11-9166 PMC3394687

[32] Willis TA, Potrata B, Ahmed M, Hewison J, Gale R, et al (2013) Understanding of and attitudes to genetic testing for inherited retinal disease: a patient perspective. Br J Ophthalmol 97: 1148–1154. 23813418 10.1136/bjophthalmol-2013-303434 PMC3756432

