# Supplementary material for: Mutation Screening of Retinal Dystrophy Patients by Targeted Capture from Tagged Pooled DNAs and Next Generation Sequencing
Source: PLoS One. 2014 Aug 18;9(8):e104281. doi: 10.1371/journal.pone.0104281 (PMC4136783; doi:10.1371/journal.pone.0104281)
Supplement: File S1 — Supplementary figure and tables. (PDF) [file pone.0104281.s001.pdf]

Supplementary Figure S1. Chromatograms for each mutation.

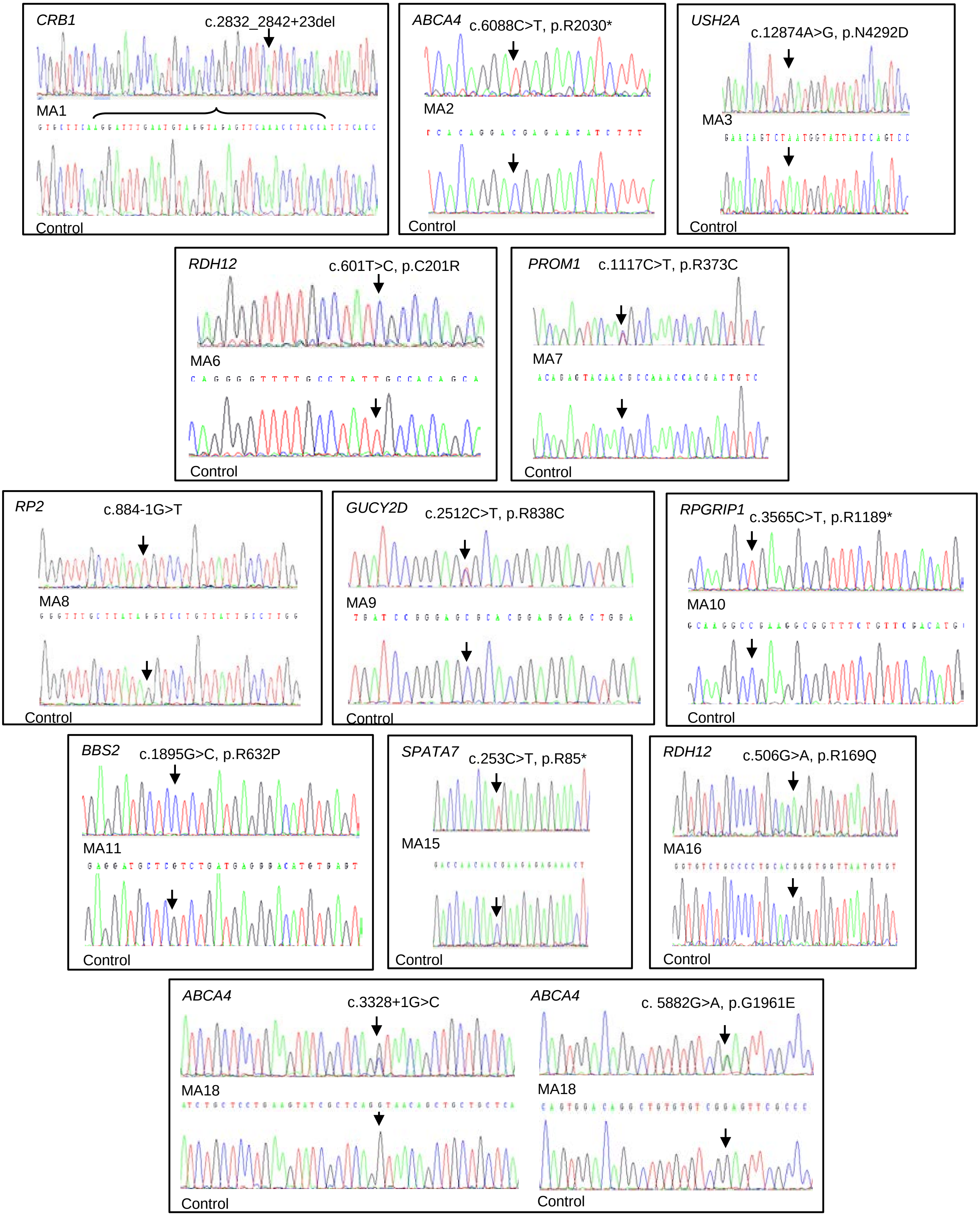

Supplementary Table S1 Summary of specific details of families that were studied.

ID	Diagnosis	Inheritance Pattern	Ethnicity	Number of Affected Cases	Number of Affected Cases Sampled	Number of Unaffected Cases Sampled
MA1	LCA	Rec.	Asian	4	2	0
MA2	CRD	Rec./Dom.	Asian	5	3	0
MA3	RP	Rec.	Asian	3	2	0
MA4	RP	Rec.	Asian	2	2	0
MA5	CRD	Dom.	European	2	2	0
MA6	RP	Rec.	Asian	2	1	0
MA7	CRD	Dom.	European	12	8	4
MA8	RP with maculopathy	Dom./X-link.	European	10	9	5
MA9	MD	Dom.	European	18	12	2
MA10	CRD	Rec.	Asian	6	6	1
MA11	RP	Rec.	European	2	2	0
MA12	CRD	Rec.	Asian	9	6	9
MA13	RP	Rec.	Asian	2	2	4
MA14	RP	Rec.	Asian	2	2	0
MA15	CRD	Rec.	Asian	4	4	7
MA16	LCA	Rec.	Asian	2	2	0
MA17	RCD	Rec.	Asian	2	2	0
MA18	CRD	Rec.	Asian	3	3	4
MA19	RCD	Rec.	Asian	8	3	3
MA20	RP	Rec.	Asian	2	2	5

The ID, diagnosis, inheritance pattern, ethnicity, number of cases and numbers sampled are shown. LCA = leber congenital amaurosis; CRD = cone rod dystrophy; RP = retinitis pigmentosa; MD = macular dystrophy and RCD = rod cone dystrophy.

Supplementary Table S2 Gene list of 162 used to generate the targeted reagent.

**ABCA4**	**CA4**	**COL11A1**	**GUCY2D**	**NDP**	**PDE6C**	**RDH12**	**SNRNP200**	**VCAN**
**ABCC6**	**CABP4**	**CRB1**	**HMCN1**	**NPHP1**	**PDZD7**	**RDH5**	**SPATA7**	**WFS1**
**ADAM9**	**CACNA1F**	**CRX**	**HTRA1**	**NPHP3**	**PEX1**	**RGR**	**TEAD1**	
**AHI1**	**CACNA2D4**	**CYP4V2**	**IDH3B**	**NPHP4**	**PEX7**	**RGS9**	**TIMM8A**	
**AIPL1**	**CC2D2A**	**DFNB31**	**IMPDH1**	**NR2E3**	**PGK1**	**RGS9BP**	**TIMP3**	
**ALMS1**	**CDH23**	**DMD**	**INPP5E**	**NRL**	**PHYH**	**RHO**	**TLR3**	
**ARL6**	**CEP290**	**EFEMP1**	**INVS**	**NYX**	**PITPNM3**	**RIMS1**	**TLR4**	
**ARMS2**	**CERKL**	**ELOVL4**	**IQCB1**	**OAT**	**PRCD**	**RLBP1**	**TMEM126A**	
**ATXN7**	**CFB**	**ERCC6**	**JAG1**	**OFD1**	**PROM1**	**ROM1**	**TOPORS**	
**BBS1**	**CFH**	**EYS**	**KCNJ13**	**OPA1**	**PRPF3**	**RP1**	**TREX1**	
**BBS2**	**CHM**	**FBLN5**	**KCNV2**	**OPA3**	**PRPF6**	**RP2**	**TRIM32**	
**BBS4**	**CLN3**	**FSCN2**	**KLHL7**	**OPN1LW**	**PRPF8**	**RP9**	**TRPM1**	
**BBS5**	**CLRN1**	**FZD4**	**LCA5**	**OPN1MW**	**PRPF31**	**RPE65**	**TSPAN12**	
**BBS7**	**CNGA1**	**GNAT1**	**LRAT**	**OPN1SW**	**PRPH2**	**RPGR**	**TTC8**	
**BBS9**	**CNGA3**	**GNAT2**	**LRP5**	**OTX2**	**PXMP3**	**RPGRIP1**	**TTPA**	
**BBS10**	**CNGB1**	**GPR98**	**MERTK**	**PANK2**	**RAX2**	**RPGRIP1L**	**TULP1**	
**BBS12**	**CNGB3**	**GRK1**	**MFRP**	**PAX2**	**RB1**	**RS1**	**UNC119**	
**BEST1**	**CNNM4**	**GRM6**	**MKKS**	**PCDH15**	**RBP3**	**SAG**	**USH1C**	
**C2**	**COL2A1**	**GUCA1A**	**MTTP**	**PDE6A**	**RBP4**	**SDCCAG8**	**USH1G**	
**C3**	**COL9A1**	**GUCA1B**	**MYO7A**	**PDE6B**	**RD3**	**SEMA4A**	**USH2A**	

Supplementary Table S3 Exons not covered in the targeted reagent.

LOCATION (hg19)	EXON
chr1:196670427-196670481	CFH terminal exon of NM_001014975.2 / ENST00000359637
chr4:16004948-16004992	PROM1 4bp terminal exon in one UCSC transcript only (non CCDS, refseq or Ensembl)
chr4:47972892-47973137	CNGA1 first exon of CCDS47050.1
chr6:66042196-66042330	EYS terminal exon of CCDS47446.1
chr10:102777320-102777392	PDZD7 terminal exon of CCDS31269.1
chr14:88881544-88881630	SPATA7 3rd exon of uc001xws.2 (non-ccds refseq or Ensembl)
chr16:53656110-53656288	RPGRIP1L exon 19 of CCDS32447.1
chrX:38144793-38146598	RPGR terminal exon of one UCSC transcript only (ORF15)
chrX:85226551-85226610	CHM terminal exon of CCDS48139.1

Supplementary Table S4 List of candidate variants in the 4 patient verification study.

	Chr	Position	Gene	Coding Effect	cDNA change	Protein change	BLOSUM62	AGVGD class	SIFTprediction	MAPPprediction	Zygosity
(A)	1	216219858	*USH2A*	missense	NM_206933.2:c.6240G>T	p.Lys2080Asn	0	C0	Tolerated	good	Het
	4	47954624	*CNGA1*	missense	NM_001142564.1:c.302G>A	p.Arg101Gln	1	C0	Tolerated	good	Het
	7	33136162	*RP9*	missense	NM_203288.1:c.410A>T	p.His137Leu	***-3***	***C15***	***Deleterious***	***bad***	Het
	8	55541086	*RP1*	missense	NM_006269.1:c.4644T>G	p.Ser1548Arg	***-1***	C0	Tolerated	***bad***	Het
	9	2718127	*KCNV2*	missense	NM_133497.3:c.388A>C	p.Thr130Pro	***-1***	C0	***Deleterious***	***bad***	Het
	9	120470884	*TLR4*	missense	NM_138554.3:c.137A>G	p.Tyr46Cys	***-2***	***C15***	Tolerated	***bad***	Het
	17	79502218	*FSCN2*	missense	NM_001077182.2:c.967G>A	p.Ala323Thr	0	C0	Tolerated	good	Het
(B)	6	11374	*C2*	splicing	NM_000063.4:c.617-5C>A	p.?	NA	NA	NA	NA	Het
	1	186045644	*HMCN1*	missense	NM_031935.2:c.8375A>G	p.Asn2792Ser	1	***C45***	***Deleterious***	***Bad***	Het
	1	197390534	*CRB1*	nonsense	NM_201253.2:c.1576C>T	p.Arg526*	NA	NA	NA	NA	Het
	1	197404300	*CRB1*	missense	NM_201253.2:c.3307G>A	p.Gly1103Arg	***-2***	***C25***	***Deleterious***	***bad***	Het
	1	215953246	*USH2A*	missense	NM_206933.2:c.10878G>T	p.Arg3626Ser	***-1***	C0	Tolerated	good	Het
	4	619426	*PDE6B*	missense	NM_000283.3:c.11G>T	p.Ser4Ile	***-2***	C0	***Deleterious***	good	Het
	6	135811814	*AHI1*	missense	NM_017651.4:c.82C>T	p.Arg28Cys	***-3***	C0	Tolerated	unknown	Het
	9	117266891	*DFNB31*	missense	NM_015404.3:c.191C>A	p.Ala64Asp	***-2***	C0	***Deleterious***	***bad***	Het
	10	102782113	*PDZD7*	missense	NM_001195263.1:c.572T>A	p.Val191Glu	***-2***	***C65***	***Deleterious***	***bad***	Het
	12	88472996	*CEP290*	missense	NM_025114.3:c.5237G>A	p.Arg1746Gln	1	C0	Tolerated	good	Het
	X	31676133	*DMD*	missense	NM_004006.2:c.8001T>A	p.Asn2667Lys	0	***C65***	***Deleterious***	***bad***	Homo
	X	38147286	*RPGR*	in-frame	NM_001034853.1:c.1579_1581delTTG	p.Gln527del	NA	NA	NA	NA	Het
(C)	1	186158843	*HMCN1*	missense	NM_031935.2:c.16741G>A	p.Ala5581Thr	0	C0	***Deleterious***	***bad***	Het
	1	216052344	*USH2A*	missense	NM_206933.2:c.8320G>A	p.Ala2774Thr	0	C0	Tolerated	good	Homo
	2	110962496	*NPHP1*	missense	NM_000272.3:c.50A>G	p.Asn17Ser	1	C0	Tolerated	good	Het
	8	55533891	*RP1*	missense	NM_006269.1:c.365G>C	p.Arg122Pro	***-2***	***C15***	***Deleterious***	***bad***	Het
	8	55538727	*RP1*	frameshift	NM_006269.1:c.2285_2289delTAAAT	p.Leu762Tyrfs*17	NA	NA	NA	NA	Het
	10	50732202	*ERCC6*	missense	NM_000124.2:c.1274A>C	p.Asp425Ala	***-2***	***C65***	***Deleterious***	***bad***	Het
	10	73270907	*CDH23*	missense	NM_022124.5:c.367G>C	p.Gly123Arg	***-2***	***C15***	***Deleterious***	***bad***	Het
	11	76891457	*MYO7A*	missense	NM_000260.3:c.2624C>G	p.Ala875Gly	0	C0	Tolerated	***bad***	Het
	16	57950041	*CNGB1*	missense	NM_001297.4:c.2209C>T	p.Arg737Cys	***-3***	C0	Tolerated	good	Het
	X	38156584	*RPGR*	missense	NM_001034853.1:c.1367A>G	p.Gln456Arg	1	C0	Tolerated	***bad***	Het
(D)	3	121500697	*IQCB1*	missense	NM_001023570.2:c.1303C>T	p.Arg435Cys	***-3***	C0	***Deleterious***	***bad***	Het
	7	128415833	*OPN1SW*	missense	NM_001708.2:c.12G>A	p.Met4Ile	1	C0	***Deleterious***	***bad***	Het
	19	54621976	*PRPF31*	frameshift	NM_015629.3:c.201delT	p.Ile67Metfs*14	NA	NA	NA	NA	Het

The diagnosis of the patients was either RP (for A, C and D) or LCA (for B). The chromosome and position of the variants are depicted according to the human genome assembly, hg19. Text in bold and italicised highlights potential pathogenic missense variants. For BLOSUM62, high pathogenicity=<0; AGVGD, high pathogenicity=C15 to C65; SIFT prediction, high pathogenicity=deleterious; MAPP prediction, high pathogenicity=bad. Homo = homozygous. Het = heterozygous.

Supplementary Table S5. List of variants in the 20 patient study after filtering (MA1-MA20)

MA1: female, family history suggests LCA and recessive inheritance

Segregates	Chr	Position	Gene	Coding Effect	cDNA change	Protein change	BLOSUM62	AGVGD class	SIFTprediction	MAPPprediction	Zygosity	Protein Domain	Reference
	1	186010250	HMCN1	missense	NM_031935.2:c.6286A>G	p.Ile2096Val	3	C0	Tolerated	good	Homo	Immunoglobulin I-set	
	1	215820993	USH2A	missense	NM_206933.2:c.14662A>T	p.Thr4888Ser	1	C0	Tolerated	good	Het	Fibronectin, type III	
	1	216405368	USH2A	missense	NM_206933.2:c.2920G>A	p.Asp974Asn	1	***C15***	***Deleterious***	***bad***	Het	EGF-like, laminin	
	1	216496929	USH2A	missense	NM_206933.2:c.1437C>A	p.Phe479Leu	0	***C15***	***Deleterious***	***bad***	Het	Laminin, N-terminal	
YES	**1**	**197398744**	**CRB1**	**frameshift**	**NM_201253.2:c.2832_2842+23del**	**p.?**	NA	NA	NA	NA	Homo		
	3	63981343	ATXN7	missense	NM_001177387.1:c.1845C>G	p.Ser615Arg	***-1***	C0	Tolerated	good	Het		
	4	122766846	BBS7	missense	NM_176824.2:c.1043A>G	p.Glu348Gly	***-2***	***C65***	***Deleterious***	***bad***	Het	BBS7 protein	
	5	89925326	GPR98	missense	NM_032119.3:c.1809C>A	p.Phe603Leu	0	***C15***	***Deleterious***	***bad***	Het		
	5	90149261	GPR98	missense	NM_032119.3:c.17365A>G	p.Lys5789Glu	1	C0	Tolerated	good	Het		
	7	33545217	BBS9	missense	NM_198428.2:c.2258A>T	p.Glu753Val	***-2***	C0	***Deleterious***	***bad***	Het		
	7	92140266	PEX1	missense	NM_000466.2:c.1579A>G	p.Thr527Ala	0	C0	Tolerated	good	Het		
	8	38869207	ADAM9	missense	NM_003816.2:c.226G>A	p.Glu76Lys	1	C0	Tolerated	good	Het	Peptidase M12B, propeptide	
	9	32542056	TOPORS	missense	NM_005802.4:c.2467A>G	p.Ser823Gly	0	C0	Tolerated	good	Het		
	9	32542166	TOPORS	missense	NM_005802.4:c.2357G>A	p.Arg786Gln	1	C0	Tolerated	good	Het		
	10	50669416	ERCC6	missense	NM_000124.2:c.3965G>T	p.Gly1322Val	***-3***	***C65***	***Deleterious***	***bad***	Het		
	10	73573082	CDH23	missense	NM_022124.5:c.9715T>C	p.Ser3239Pro	***-1***	C0	***Deleterious***	***bad***	Het		
	15	31342673	TRPM1	missense	NM_002420.4:c.1310G>T	p.Gly437Val	***-3***	C0	***Deleterious***	***bad***	Homo		
	X	49076224	CACNA1F	In-frame	NM_005183.2:c.2439_2444dupTCCTCC	p.Glu824_Glu825dup	NA	NA	NA	NA	Het		

Segregates	Chr	Position	Gene	Coding Effect	cDNA change	Protein change	BLOSUM62	AGVGD class	SIFTprediction	MAPPprediction	Zygosity	Protein Domain	Reference
**MA2: male, family history suggests CRD and recessive or dominant inheritance**													
	1	5925272	NPHP4	missense	NM_015102.3:c.3706G>A	p.Val1236Met	1	C0	***Deleterious***	***bad***	Het		
YES	**1**	**94471056**	**ABCA4**	**nonsense**	**NM_000350.2:c.6088C>T**	**p.Arg2030***	NA	NA	NA	NA	Homo	ABC transporter-like	[19]
	1	186120461	HMCN1	missense	NM_031935.2:c.14738C>A	p.Thr4913Asn	0	C0	***Deleterious***	***bad***	Het	G2 nidogen/fibulin G2F	
	1	215914751	USH2A	missense	NM_206933.2:c.11677C>A	p.Pro3893Thr	***-1***	***C35***	***Deleterious***	***bad***	Het	Fibronectin, type III	
	2	96950323	SNRNP200	missense	NM_014014.4:c.4165G>A	p.Val1389Ile	3	C0	Tolerated	good	Het	DEAD/DEAH box type, N-terminal	
	4	15554873	CC2D2A	missense	NM_001080522.2:c.2431G>A	p.Glu811Lys	1	C0	***Deleterious***	unknown	Het		
	6	65596607	EYS	missense	NM_001142800.1:c.2975G>T	p.Cys992Phe	***-2***	C0	***Deleterious***	unknown	Het	Epidermal growth factor-like, type 3	
	10	73405717	CDH23	missense	NM_022124.5:c.1270G>A	p.Val424Met	1	C0	***Deleterious***	***bad***	Het	Cadherin	
	12	76740134	BBS10	missense	NM_024685.3:c.1631A>G	p.Asn544Ser	1	C0	Tolerated	good	Het		
	12	88480262	CEP290	missense	NM_025114.3:c.4208G>C	p.Arg1403Thr	***-1***	C0	Tolerated	good	Het		
**MA3: female, family history suggests RP and recessive inheritance**													
	6	137193331	PEX7	splicing?	NM_000288.3:c.748-5dupT	p.?	NA	NA	NA	NA	Het		
	1	68910315	RPE65	missense	NM_000329.2:c.394G>A	p.Ala132Thr	0	C0	Tolerated	good	Het	Carotenoid oxygenase	
YES	**1**	**215848379**	**USH2A**	**missense**	**NM_206933.2:c.12874A>G**	**p.Asn4292Asp**	1	***C15***	***Deleterious***	***bad***	Homo	Fibronectin, type III	
	9	120475185	TLR4	missense	NM_138554.3:c.779T>C	p.Leu260Pro	***-3***	***C25***	***Deleterious***	***bad***	Het	Toll-like receptor	
	9	139333568	INPP5E	missense	NM_019892.3:c.304G>T	p.Asp102Tyr	***-3***	C0	***Deleterious***	good	Het		
	10	73537449	CDH23	missense	NM_022124.5:c.4858G>A	p.Val1620Met	1	***C15***	***Deleterious***	***bad***	Het	Cadherin	
	11	66291004	BBS1	missense	NM_024649.4:c.908T>C	p.Val303Ala	0	***C25***	***Deleterious***	good	Het	WD40 repeat-like domain	
	16	56536660	BBS2	missense	NM_031885.3:c.865A>G	p.Ile289Val	3	C0	Tolerated	good	Het	BBS2 protein	
	X	13774746	OFD1	missense	NM_003611.2:c.1271A>G	p.Asn424Ser	1	C0	***Deleterious***	good	Het		

Segregates	Chr	Position	Gene	Coding Effect	cDNA change	Protein change	BLOSUM62	AGVGD class	SIFTprediction	MAPPprediction	Zygosity	Protein Domain	Reference
**MA4: male, family history suggests RP and recessive inheritance**													
	1	5969225	NPHP4	missense	NM_015102.3:c.1490C>G	p.Pro497Arg	***-2***	C0	***Deleterious***	***bad***	Het		
	1	94544183	ABCA4	missense	NM_000350.2:c.1319A>G	p.Tyr440Cys	***-2***	***C15***	Tolerated	good	Het	Rim ABC transporter	
	1	197297965	CRB1	missense	NM_201253.2:c.484G>A	p.Val162Met	1	C0	Tolerated	good	Het	EGF	
	1	215848645	USH2A	missense	NM_206933.2:c.12608A>G	p.Gln4203Arg	1	C0	Tolerated	good	Het	Fibronectin, type III	
	4	123663048	BBS12	start loss	NM_152618.2:c.1A>C	p.?	NA	NA	NA	NA	Het		
	4	187118692	CYP4V2	missense	NM_207352.3:c.610G>A	p.Ala204Thr	0	C0	***Deleterious***	***bad***	Het	Cytochrome P450	
YES	6	64498971	EYS	missense	NM_001142800.1:c.7558T>C	p.Phe2520Leu	0	C0	Tolerated	unknown	Homo	Laminin G, subdomain 2	
YES	6	66204970	EYS	missense	NM_001142800.1:c.334G>A	p.Val112Ile	3	C0	Tolerated	good	Homo		
	6	135726089	AHI1	frameshift	NM_017651.4:c.2988delT	p.Val997Serfs*20	NA	NA	NA	NA	Het		
	8	87645092	CNGB3	missense	NM_019098.4:c.1208G>A	p.Arg403Gln	1	C0	***Deleterious***	***bad***	Het		
	9	103059231	INVS	missense	NM_014425.2:c.2819G>A	p.Arg940Gln	1	C0	Tolerated	good	Het	IQ motif, EF-hand binding site	
	10	73537449	CDH23	missense	NM_022124.5:c.4858G>A	p.Val1620Met	1	***C15***	***Deleterious***	***bad***	Het	Cadherin	
	11	76893481	MYO7A	missense	NM_000260.3:c.3121G>T	p.Val1041Phe	***-1***	C0	***Deleterious***	good	Het	MyTH4 domain	
	12	48371141	COL2A1	missense	NM_001844.4:c.3235G>A	p.Ala1079Thr	0	C0	Tolerated	good	Het		
	15	12084	NR2E3	missense	NM_014249.2:c.1186G>A	p.Gly396Arg	***-2***	***C65***	***Deleterious***	unknown	Het	Retinoid X receptor	
	17	63221207	RGS9	missense	NM_003835.3:c.1495T>C	p.Ser499Pro	***-1***	C0	***Deleterious***	unknown	Het		
	17	72916365	USH1G	missense	NM_173477.2:c.566G>A	p.Arg189Gln	1	C0	Tolerated	good	Het		
	17	79502218	FSCN2	missense	NM_001077182.2:c.967G>A	p.Ala323Thr	0	C0	Tolerated	good	Het	Fascin domain	
	X	32509447	DMD	missense	NM_004006.2:c.2569C>T	p.Pro857Ser	***-1***	C0	Tolerated	good	Homo	Spectrin repeat	

Segregates	Chr	Position	Gene	Coding Effect	cDNA change	Protein change	BLOSUM62	AGVGD class	SIFTprediction	MAPPprediction	Zygosity	Protein Domain	Reference
**MA5: female, family history suggests CRD and dominant inheritance**													
	1	215844373	USH2A	missense	NM_206933.2:c.14074G>A	p.Gly4692Arg	***-2***	***C65***	***Deleterious***	***bad***	Het	Fibronectin, type III	
	2	96942978	SNRNP200	missense	NM_014014.4:c.5933G>C	p.Gly1978Ala	0	C0	Tolerated	good	Het	Sec63 domain	
	10	13336486	PHYH	missense	NM_006214.3:c.356C>T	p.Thr119Met	***-1***	C0	***Deleterious***	***bad***	Het	Phytanoyl-CoA dioxygenase	
	16	16284103	ABCC6	missense	NM_001171.5:c.1553G>A	p.Arg518Gln	1	C0	Tolerated	good	Het	transmembrane domain	
	16	53653005	RPGRIP1L	missense	NM_015272.2:c.3548C>G	p.Ala1183Gly	0	C0	***Deleterious***	***bad***	Homo		
	X	49076224	CACNA1F	In-frame	NM_005183.2:c.2442_2444dupTCC	p.Glu825dup	NA	NA	NA	NA	Het		
**MA6: male, family history suggests RP and recessive inheritance**													
	4	15982163	PROM1	splicing?	NM_006017.2:c.2374-4dupG	p.?	NA	NA	NA	NA	Homo		
	1	186007997	HMCN1	missense	NM_031935.2:c.5888G>T	p.Gly1963Val	***-3***	C0	Tolerated	good	Het	Immunoglobulin I-set	
	5	82836537	VCAN	missense	NM_004385.4:c.7715C>T	p.Ser2572Leu	***-2***	***C65***	***Deleterious***	***bad***	Het		
	10	86008700	RGR	missense	NM_002921.3:c.271G>A	p.Gly91Ser	0	***C55***	***Deleterious***	good	Het	GPCR, rhodopsin-like, 7TM	
R	**14**	**68193850**	**RDH12**	**missense**	**NM_152443.2:c.601T>C**	**p.Cys201Arg**	***-3***	C0	***Deleterious***	***bad***	Homo	Short-chain dehydrogenase/reductase SDR	[21]
	15	73029831	BBS4	missense	NM_033028.3:c.1463C>A	p.Thr488Lys	***-1***	C0	Tolerated	good	Het		
**MA7: male, family history suggests CRD and dominant inheritance**													
	6	66063346	EYS	splicing?	NM_001142800.1:c.1459+5C>T	p.?	NA	NA	NA	NA	Het		
	2	71134	ALMS1	missense	NM_015120.4:c.5362A>G	p.Asn1788Asp	1	C0	Tolerated	unknown	Het		
	2	112722801	MERTK	missense	NM_006343.2:c.791C>G	p.Ala264Gly	0	***C55***	***Deleterious***	***bad***	Het	Immunoglobulin-like	
YES	**4**	**16014922**	**PROM1**	**missense**	**NM_006017.2:c.1117C>T**	**p.Arg373Cys**	***-3***	C0	***Deleterious***	***bad***	Het	Prominin	[22,23]
	5	178413684	GRM6	missense	NM_000843.3:c.1571C>T	p.Pro524Leu	***-3***	***C65***	***Deleterious***	***bad***	Het	GPCR, family 3, nine cysteines domain	
	16	53692694	RPGRIP1L	missense	NM_015272.2:c.1340T>C	p.Leu447Ser	***-2***	C0	Tolerated	good	Het		

Segregates	Chr	Position	Gene	Coding Effect	cDNA change	Protein change	BLOSUM62	AGVGD class	SIFTprediction	MAPPprediction	Zygosity	Protein Domain	Reference
**MA8: male, family history suggests RP with maculopathy and dominant or X-linked inheritance**													
	4	15982163	PROM1	splicing?	NM_006017.2:c.2374-4dupG	p.?	NA	NA	NA	NA	Het		
	X	18674770	RS1	splicing?	NM_000330.3:c.184+3G>T	p.?	NA	NA	NA	NA	Het		
YES	**X**	**46736939**	**RP2**	**splicing**	**NM_006915.2:c.884-1G>T**	**p.?**	NA	NA	NA	NA	Homo		
	1	103400669	COL11A1	missense	NM_080629.2:c.3475G>A	p.Gly1159Ser	0	***C55***	***Deleterious***	good	Het		
	1	215844373	USH2A	missense	NM_206933.2:c.14074G>A	p.Gly4692Arg	***-2***	***C65***	***Deleterious***	***bad***	Het	Fibronectin, type III	
	5	89969880	GPR98	missense	NM_032119.3:c.4939A>G	p.Ile1647Val	3	C0	Tolerated	good	Het	Na-Ca exchanger/integrin-beta4	
	10	13320305	PHYH	in-frame	NM_006214.3:c.1010_1012dupGAT	p.Asn337_Leu338insHis	NA	NA	NA	NA	Het		
	11	119216627	MFRP	missense	NM_031433.2:c.283G>A	p.Ala95Thr	0	C0	Tolerated	good	Het		
	15	31294159	TRPM1	missense	NM_002420.4:c.4678G>A	p.Val1560Met	1	C0	***Deleterious***	good	Het		
NO	15	6267	NR2E3	nonsense	NM_014249.2:c.300C>A	p.Cys100*	NA	NA	NA	NA	Het	Zinc finger, nuclear hormone receptor-type	
A	X	38182144	RPGR	missense	NM_001034853.1:c.209G>A	p.Gly70Glu	***-2***	C0	***Deleterious***	***bad***	Het	Regulator of chromosome condensation, RCC1	
**MA9: female, family history suggests Macular dystrophy and dominant inheritance**													
	4	15982163	PROM1	splicing?	NM_006017.2:c.2374-4dupG	p.?	NA	NA	NA	NA	Homo		
	16	53683031	RPGRIP1L	splicing?	NM_015272.2:c.2153-4G>C	p.?	NA	NA	NA	NA	Het		
NO	1	186141213	HMCN1	missense	NM_031935.2:c.15764T>C	p.Ile5255Thr	***-1***	***C65***	***Deleterious***	***bad***	Het	EGF-like calcium-binding	
	1	216172258	USH2A	missense	NM_206933.2:c.6628C>G	p.Pro2210Ala	***-1***	***C25***	***Deleterious***	***bad***	Het	Fibronectin, type III	
	16	53653005	RPGRIP1L	missense	NM_015272.2:c.3548C>G	p.Ala1183Gly	0	C0	***Deleterious***	***bad***	Het		
YES	**17**	**7918018**	**GUCY2D**	**missense**	**NM_000180.3:c.2512C>T**	**p.Arg838Cys**	***-3***	***C65***	***Deleterious***	***bad***	Het	Haem NO binding associated	[24,25]
	20	10393439	MKKS	missense	NM_170784.1:c.724G>T	p.Ala242Ser	1	***C15***	***Deleterious***	good	Het	Chaperonin Cpn60/TCP-1	

Segregates	Chr	Position	Gene	Coding Effect	cDNA change	Protein change	BLOSUM62	AGVGD class	SIFTprediction	MAPPprediction	Zygosity	Protein Domain	Reference
**MA10: female, family history suggests CRD and recessive inheritance**													
	1	215960153	USH2A	missense	NM_206933.2:c.10246T>G	p.Cys3416Gly	***-3***	***C65***	***Deleterious***	***bad***	Het	Fibronectin, type III	
	1	216062306	USH2A	missense	NM_206933.2:c.7685T>C	p.Val2562Ala	0	C0	Tolerated	good	Het	Fibronectin, type III	
	2	96942928	SNRNP200	missense	NM_014014.4:c.5983G>A	p.Ala1995Thr	0	C0	Tolerated	good	Het	Sec63 domain	
	5	82835550	VCAN	missense	NM_004385.4:c.6728C>G	p.Thr2243Arg	***-1***	C0	Tolerated	good	Het		
	9	139326278	INPP5E	missense	NM_019892.3:c.1547A>G	p.Lys516Arg	2	C0	Tolerated	good	Het	Endonuclease/exonuclease/phosphatase	
	10	73337684	CDH23	missense	NM_022124.5:c.767G>A	p.Arg256His	0	C0	***Deleterious***	***bad***	Het	Cadherin	
	11	17531058	USH1C	missense	NM_153676.3:c.1858C>T	p.Arg620Cys	***-3***	C0	***Deleterious***	unknown	Het		
	12	2022196	CACNA2D4	missense	NM_172364.4:c.419C>G	p.Ala140Gly	0	C0	***Deleterious***	***good***	Het		
YES	**14**	**21813304**	**RPGRIP1**	**nonsense**	**NM_020366.3:c.3565C>T**	**p.Arg1189***	NA	NA	NA	NA	Homo	-	[26]
	15	73028295	BBS4	missense	NM_033028.3:c.1236A>T	p.Glu412Asp	2	C0	Tolerated	good	Het		
	16	53698905	RPGRIP1L	missense	NM_015272.2:c.1120C>T	p.His374Tyr	2	***C15***	***Deleterious***	good	Het		
	17	79503621	FSCN2	missense	NM_001077182.2:c.1151C>A	p.Ala384Glu	***-1***	C0	Not scored	unknown	Het		

MA11: male, family history suggests RP and recessive inheritance

Segregates	Chr	Position	Gene	Coding Effect	cDNA change	Protein change	BLOSUM62	AGVGD class	SIFTprediction	MAPPprediction	Zygosity	Protein Domain	Reference
	1	6012898	NPHP4	splicing	NM_015102.3:c.674-2A>G	p.?	NA	NA	NA	NA	Het		
	1	94467548	ABCA4	missense	NM_000350.2:c.6148G>C	p.Val2050Leu	1	***C25***	***Deleterious***	***bad***	Het	ABC transporter-like	
	1	216011442	USH2A	missense	NM_206933.2:c.9262G>A	p.Glu3088Lys	1	***C55***	***Deleterious***	***bad***	Het	Fibronectin, type III	
	4	16077349	PROM1	missense	NM_006017.2:c.181A>G	p.Ile61Val	3	C0	Tolerated	good	Het	Prominin	
	6	42153428	GUCA1B	missense	NM_002098.5:c.465G>T	p.Glu155Asp	2	C0	Tolerated	good	Het	EF-HAND 2	
	6	80626456	ELOVL4	missense	NM_022726.3:c.814G>C	p.Glu272Gln	2	C0	Tolerated	good	Het	GNS1/SUR4 membrane protein	
	9	2718127	KCNV2	missense	NM_133497.3:c.388A>C	p.Thr130Pro	***-1***	C0	***Deleterious***	***bad***	Het	Potassium channel, voltage dependent, Kv, tetramerisation	
	9	120476583	TLR4	missense	NM_138554.3:c.2177G>T	p.Gly726Val	***-3***	***C65***	***Deleterious***	***bad***	Het	Toll/interleukin-1 receptor homology domain	
	11	76883864	MYO7A	missense	NM_000260.3:c.1868G>A	p.Arg623His	0	C0	***Deleterious***	good	Het	Myosin head, motor domain	
	12	1908849	CACNA2D4	missense	NM_172364.4:c.2987T>C	p.Phe996Ser	***-2***	C0	Tolerated	unknown	Het		
	16	53686789	RPGRIP1L	missense	NM_015272.2:c.1810G>A	p.Glu604Lys	1	C0	***Deleterious***	***bad***	Het	Protein of unknown function DUF3250	
YES	**16**	**56530894**	**BBS2**	**missense**	**NM_031885.3:c.1895G>C**	**p.Arg632Pro**	***-2***	***C15***	Tolerated	***bad***	Homo	BBS2 protein	[27]
A	X	49082958	CACNA1F	missense	NM_005183.2:c.1409T>C	p.Leu470Pro	***-3***	C0	***Deleterious***	***bad***	Het		

Segregates	Chr	Position	Gene	Coding Effect	cDNA change	Protein change	BLOSUM62	AGVGD class	SIFTprediction	MAPPprediction	Zygosity	Protein Domain	Reference
**MA12: male, family history suggests CRD and recessive inheritance**													
	4	15982163	PROM1	splicing?	NM_006017.2:c.2374-4dupG	p.?	NA	NA	NA	NA	Homo		
	16	56534761	BBS2	splicing?	NM_031885.3:c.1397+5C>G	p.?	NA	NA	NA	NA	Het		
	6	5304	CFB	missense	NM_001710.5:c.26T>A	p.Leu9His	***-3***	C0	***Deleterious***	***bad***	Het	Complement B/C2	
	9	2718127	KCNV2	missense	NM_133497.3:c.388A>C	p.Thr130Pro	***-1***	C0	***Deleterious***	***bad***	Het	Potassium channel, voltage dependent, Kv, tetramerisation	
NO	10	73553052	CDH23	missense	NM_022124.5:c.6367G>A	p.Gly2123Arg	***-2***	***C65***	***Deleterious***	***bad***	Het	Cadherin	
NO	10	73563067	CDH23	missense	NM_022124.5:c.7762G>C	p.Glu2588Gln	2	C0	Tolerated	good	Het	Cadherin	
	12	76740134	BBS10	missense	NM_024685.3:c.1631A>G	p.Asn544Ser	1	C0	Tolerated	good	Het		
	12	88454728	CEP290	missense	NM_025114.3:c.6401T>C	p.Ile2134Thr	***-1***	C0	***Deleterious***	***bad***	Het		
	17	63221207	RGS9	missense	NM_003835.3:c.1495T>C	p.Ser499Pro	***-1***	C0	***Deleterious***	unknown	Het		
	17	72916338	USH1G	missense	NM_173477.2:c.593A>C	p.His198Pro	***-2***	C0	Tolerated	***bad***	Het		
	20	10625830	JAG1	missense	NM_000214.2:c.2188A>G	p.Met730Val	1	C0	Tolerated	***bad***	Het	EGF, extracellular	
**MA13: female, family history suggests RP and recessive inheritance**													
	4	100503136	MTTP	missense	NM_000253.2:c.136C>G	p.Arg46Gly	***-2***	C0	Tolerated	good	Het	Lipid transport protein, N-terminal	
NO	5	89924514	GPR98	missense	NM_032119.3:c.1374T>A	p.Phe458Leu	0	***C15***	***Deleterious***	***bad***	Het		
A	5	90149261	GPR98	missense	NM_032119.3:c.17365A>G	p.Lys5789Glu	1	C0	Tolerated	good	Het		
	9	2718127	KCNV2	missense	NM_133497.3:c.388A>C	p.Thr130Pro	***-1***	C0	***Deleterious***	***bad***	Het	Potassium channel, voltage dependent, Kv, tetramerisation	
	9	117266942	DFNB31	missense	NM_015404.3:c.140C>A	p.Thr47Asn	0	C0	Tolerated	good	Het		
	11	76891457	MYO7A	missense	NM_000260.3:c.2624C>G	p.Ala875Gly	0	C0	Tolerated	***bad***	Het		
	11	76910630	MYO7A	missense	NM_000260.3:c.4619C>T	p.Ala1540Val	0	C0	Tolerated	unknown	Het	FERM domain	

Segregates	Chr	Position	Gene	Coding Effect	cDNA change	Protein change	BLOSUM62	AGVGD class	SIFTprediction	MAPPprediction	Zygosity	Protein Domain	Reference

MA14: male, family history suggests RP and recessive inheritance

Segregates	Chr	Position	Gene	Coding Effect	cDNA change	Protein change	BLOSUM62	AGVGD class	SIFTprediction	MAPPprediction	Zygosity	Protein Domain	Reference
	6	137193331	PEX7	splicing?	NM_000288.3:c.748-5dupT	p.?	NA	NA	NA	NA	Het		
	4	15517532	CC2D2A	missense	NM_001080522.2:c.922T>C	p.Phe308Leu	0	C0	Tolerated	good	Het		
A	4	123664710	BBS12	missense	NM_152618.2:c.1663G>A	p.Glu555Lys	1	C0	Tolerated	good	Het	Chaperonin Cpn60/TCP-1	
YES	4	123665061	BBS12	missense	NM_152618.2:c.2014G>A	p.Ala672Thr	0	***C55***	***Deleterious***	***bad***	Het		
	5	178418549	GRM6	missense	NM_000843.3:c.733A>G	p.Ile245Val	3	***C15***	Tolerated	***bad***	Homo	Extracellular ligand-binding receptor	
	6	72892193	RIMS1	missense	NM_014989.4:c.1019C>T	p.Ala340Val	0	C0	Tolerated	good	Het		
	9	2718127	KCNV2	missense	NM_133497.3:c.388A>C	p.Thr130Pro	***-1***	C0	***Deleterious***	***bad***	Het	Potassium channel, voltage dependent, Kv, tetramerisation	
	11	66283020	BBS1	missense	NM_024649.4:c.442G>A	p.Asp148Asn	1	C0	Tolerated	good	Het		
	11	119216338	MFRP	missense	NM_031433.2:c.433G>A	p.Gly145Arg	***-2***	***C25***	***Deleterious***	***bad***	Het	CUB	
	16	53705492	RPGRIP1L	missense	NM_015272.2:c.1033C>A	p.Gln345Lys	1	C0	Tolerated	good	Het		
	17	63221207	RGS9	missense	NM_003835.3:c.1495T>C	p.Ser499Pro	***-1***	C0	***Deleterious***	unknown	Het		
A	17	79495853	FSCN2	missense	NM_001077182.2:c.296G>T	p.Arg99Leu	***-2***	C0	***Deleterious***	***bad***	Het	Fascin domain	

Segregates	Chr	Position	Gene	Coding Effect	cDNA change	Protein change	BLOSUM62	AGVGD class	SIFTprediction	MAPPprediction	Zygosity	Protein Domain	Reference

MA15: male, family history suggests CRD and recessive inheritance

Segregates	Chr	Position	Gene	Coding Effect	cDNA change	Protein change	BLOSUM62	AGVGD class	SIFTprediction	MAPPprediction	Zygosity	Protein Domain	Reference
	1	94473807	ABCA4	missense	NM_000350.2:c.5882G>A	p.Gly1961Glu	***-2***	***C65***	***Deleterious***	***bad***	Het	ABC transporter-like	
	5	82817313	VCAN	missense	NM_004385.4:c.3188T>C	p.Leu1063Pro	***-3***	C0	***Deleterious***	***bad***	Homo		
	5	89948189	GPR98	missense	NM_032119.3:c.3443G>A	p.Gly1148Asp	***-1***	***C65***	***Deleterious***	***bad***	Homo		
	6	64431505	EYS	missense	NM_001142800.1:c.8422G>A	p.Ala2808Thr	0	C0	***Deleterious***	unknown	Het	Laminin G, subdomain 2	
	6	65300160	EYS	missense	NM_001142800.1:c.5600C>T	p.Ser1867Phe	***-2***	C0	***Deleterious***	unknown	Het		
	8	55538820	RP1	missense	NM_006269.1:c.2378G>T	p.Arg793Ile	***-3***	***C15***	***Deleterious***	***bad***	Het		
	9	117266673	DFNB31	missense	NM_015404.3:c.409G>C	p.Glu137Gln	2	C0	Tolerated	good	Het		
	9	139333403	INPP5E	missense	NM_019892.3:c.469G>T	p.Gly157Trp	***-2***	C0	Tolerated	good	Het		
	11	66291279	BBS1	missense	NM_024649.4:c.1036G>A	p.Val346Ile	3	***C25***	***Deleterious***	***bad***	Het	WD40 repeat-like-containing domain	
	11	68115675	LRP5	missense	NM_002335.2:c.452A>C	p.Asp151Ala	***-2***	***C65***	***Deleterious***	***bad***	Het	LDLR class B repeat	
	11	76891457	MYO7A	missense	NM_000260.3:c.2624C>G	p.Ala875Gly	0	C0	Tolerated	***bad***	Het		
	11	76891460	MYO7A	missense	NM_000260.3:c.2627A>G	p.Glu876Gly	***-2***	C0	***Deleterious***	***bad***	Het		
	12	1906632	CACNA2D4	missense	NM_172364.4:c.3065C>T	p.Pro1022Leu	***-3***	C0	Tolerated	unknown	Het		
YES	**14**	**88883069**	**SPATA7**	**nonsense**	**NM_018418.4:c.253C>T**	**p.Arg85***	NA	NA	NA	NA	Homo	-	[28]
	17	63221207	RGS9	missense	NM_003835.3:c.1495T>C	p.Ser499Pro	***-1***	C0	***Deleterious***	unknown	Het		
	20	10622214	JAG1	missense	NM_000214.2:c.2810G>A	p.Arg937Gln	1	C0	Tolerated	good	Het		

Segregates	Chr	Position	Gene	Coding Effect	cDNA change	Protein change	BLOSUM62	AGVGD class	SIFTprediction	MAPPprediction	Zygosity	Protein Domain	Reference
**MA16: male, family history suggests LCA and recessive inheritance**													
	1	186072648	HMCN1	missense	NM_031935.2:c.10618G>A	p.Val3540Ile	3	***C25***	***Deleterious***	***bad***	Het	Immunoglobulin I-set	
	2	110187	ALMS1	missense	NM_015120.4:c.8983G>A	p.Val2995Ile	3	C0	Tolerated	unknown	Het		
	2	169521	ALMS1	missense	NM_015120.4:c.9917A>G	p.Asn3306Ser	1	C0	***Deleterious***	unknown	Het		
	3	121518211	IQCB1	missense	NM_001023570.2:c.598C>A	p.Leu200Ile	2	C0	Tolerated	good	Het		
	3	132423114	NPHP3	missense	NM_153240.4:c.1452A>G	p.Ile484Met	1	C0	***Deleterious***	good	Het		
	5	90149261	GPR98	missense	NM_032119.3:c.17365A>G	p.Lys5789Glu	1	C0	Tolerated	good	Het		
	5	178418555	GRM6	missense	NM_000843.3:c.727G>T	p.Val243Phe	***-1***	C0	Tolerated	good	Het	Extracellular ligand-binding receptor	
	6	64431505	EYS	missense	NM_001142800.1:c.8422G>A	p.Ala2808Thr	0	C0	***Deleterious***	unknown	Het	Laminin G, subdomain 2	
	7	92157661	PEX1	missense	NM_000466.2:c.89A>C	p.His30Pro	***-2***	C0	Tolerated	***bad***	Het	Peroxisome biogenesis factor 1, alpha/beta	
	8	38880817	ADAM9	missense	NM_003816.2:c.887G>A	p.Arg296Gln	1	C0	Tolerated	good	Het	Peptidase M12B, ADAM/reprolysin	
	10	95400223	PDE6C	missense	NM_006204.3:c.1646T>C	p.Met549Thr	***-1***	***C45***	***Deleterious***	***bad***	Het		
	10	95405722	PDE6C	missense	NM_006204.3:c.1853C>T	p.Thr618Met	***-1***	C0	Tolerated	good	Het	3'5'-cyclic nucleotide phosphodiesterase, catalytic domain	
	11	76891457	MYO7A	missense	NM_000260.3:c.2624C>G	p.Ala875Gly	0	C0	Tolerated	bad	Het		
YES	**14**	**68193755**	**RDH12**	**missense**	**NM_152443.2:c.506G>A**	**p.Arg169Gln**	1	***C35***	***Deleterious***	***bad***	Homo	Short-chain dehydrogenase/reductase SDR	[29]
	15	31325130	TRPM1	missense	NM_002420.4:c.2648A>G	p.Glu883Gly	***-2***	C0	***Deleterious***	***bad***	Het	Ion transport	
	16	16295863	ABCC6	missense	NM_001171.5:c.1171A>G	p.Arg391Gly	***-2***	C0	***Deleterious***	***bad***	Het	transmembrane domain	
	17	63221207	RGS9	missense	NM_003835.3:c.1495T>C	p.Ser499Pro	***-1***	C0	***Deleterious***	unknown	Het		
	20	10389422	MKKS	missense	NM_170784.1:c.1015A>G	p.Ile339Val	3	C0	Tolerated	good	Het	Chaperonin Cpn60/TCP-1	

Segregates	Chr	Position	Gene	Coding Effect	cDNA change	Protein change	BLOSUM62	AGVGD class	SIFTprediction	MAPPprediction	Zygosity	Protein Domain	Reference

MA17: male, family history suggests RCD and recessive inheritance

Segregates	Chr	Position	Gene	Coding Effect	cDNA change	Protein change	BLOSUM62	AGVGD class	SIFTprediction	MAPPprediction	Zygosity	Protein Domain	Reference
	1	5969225	NPHP4	missense	NM_015102.3:c.1490C>G	p.Pro497Arg	***-2***	C0	***Deleterious***	***bad***	Het		
	1	185976299	HMCN1	missense	NM_031935.2:c.4515C>G	p.Asp1505Glu	2	C0	Tolerated	good	Het	Immunoglobulin I-set	
	1	216348809	USH2A	missense	NM_206933.2:c.4412G>C	p.Arg1471Thr	***-1***	C0	Tolerated	good	Het		
	1	243652316	SDCCAG8	missense	NM_006642.3:c.1986G>T	p.Arg662Ser	***-1***	C0	***Deleterious***	***bad***	Het		
	5	82816753	VCAN	missense	NM_004385.4:c.2628T>A	p.His876Gln	0	C0	***Deleterious***	***bad***	Het		
	5	90024663	GPR98	missense	NM_032119.3:c.10339G>A	p.Glu3447Lys	1	C0	Tolerated	good	Het	EAR	
	7	92147143	PEX1	missense	NM_000466.2:c.686A>G	p.Asn229Ser	1	C0	Tolerated	unknown	Het		
	9	2718127	KCNV2	missense	NM_133497.3:c.388A>C	p.Thr130Pro	***-1***	C0	***Deleterious***	***bad***	Het	Potassium channel, voltage dependent, Kv, tetramerisation	
	9	120475128	TLR4	missense	NM_138554.3:c.722A>G	p.Asn241Ser	1	C0	Tolerated	good	Het	Toll-like receptor	
	10	102568919	PAX2	missense	ENST00000370296.1:c.914C>T	p.Ser305Leu	***-2***	C0	Tolerated	good	Het	Paired-box protein 2 C-terminal	
	11	86662343	FZD4	missense	NM_012193.3:c.1455G>T	p.Leu485Phe	0	C0	***Deleterious***	***bad***	Het	Frizzled protein	
	12	88472996	CEP290	missense	NM_025114.3:c.5237G>A	p.Arg1746Gln	1	C0	Tolerated	good	Het		
	X	13765025	OFD1	missense	NM_003611.2:c.781G>A	p.Val261Ile	3	C0	Tolerated	good	Homo		

MA18: female, family history suggests CRD and recessive inheritance

Segregates	Chr	Position	Gene	Coding Effect	cDNA change	Protein change	BLOSUM62	AGVGD class	SIFTprediction	MAPPprediction	Zygosity	Protein Domain	Reference
YES	**1**	**94508316**	**ABCA4**	**splicing**	**NM_000350.2:c.3328+1G>C**	**p.?**	NA	NA	NA	NA	Het		
	4	15982163	PROM1	splicing?	NM_006017.2:c.2374-4dupG	p.?	NA	NA	NA	NA	Het		
	1	6007259	NPHP4	missense	NM_015102.3:c.1024C>T	p.Arg342Cys	***-3***	C0	***Deleterious***	***bad***	Het		
	1	68904660	RPE65	missense	NM_000329.2:c.963T>G	p.Asn321Lys	0	C0	***Deleterious***	good	Het	Carotenoid oxygenase	
YES	**1**	**94473807**	**ABCA4**	**missense**	**NM_000350.2:c.5882G>A**	**p.Gly1961Glu**	***-2***	***C65***	***Deleterious***	***bad***	Het	ABC transporter-like	[30,31]
	2	96950323	SNRNP200	missense	NM_014014.4:c.4165G>A	p.Val1389Ile	3	C0	Tolerated	good	Het	DEAD/DEAH box type, N-terminal	
	6	14945	C2	missense	NM_000063.4:c.1103G>A	p.Arg368Gln	1	***C35***	***Deleterious***	***bad***	Het	von Willebrand factor, type A	
	10	48389479	RBP3	missense	NM_002900.2:c.1399C>T	p.Pro467Ser	***-1***	C0	***Deleterious***	***bad***	Het	Interphotoreceptor retinol-binding	
	10	48389841	RBP3	missense	NM_002900.2:c.1037G>A	p.Arg346His	0	C0	***Deleterious***	***bad***	Het		
	10	73551036	CDH23	missense	NM_022124.5:c.6197G>A	p.Arg2066Gln	1	C0	Tolerated	good	Het	Cadherin	
	12	76742114	BBS10	missense	NM_024685.3:c.25G>T	p.Gly9Trp	***-2***	C0	Tolerated	unknown	Het		
	12	88508951	CEP290	frameshift	NM_025114.3:c.1833delA	p.Leu612Phefs*5	NA	NA	NA	NA	Het		
	14	89307227	TTC8	missense	NM_144596.2:c.284A>G	p.Lys95Arg	2	C0	Tolerated	good	Het		
	15	31318408	TRPM1	missense	NM_002420.4:c.3497A>T	p.His1166Leu	***-3***	C0	Tolerated	good	Het		
	17	6329946	AIPL1	missense	NM_014336.3:c.773G>A	p.Arg258Gln	1	C0	Tolerated	good	Het	Tetratricopeptide repeat-containing	
	20	10393438	MKKS	missense	NM_170784.1:c.725C>T	p.Ala242Val	0	C0	Tolerated	good	Het	Chaperonin Cpn60/TCP-1	
	X	31462606	DMD	missense	NM_004006.2:c.9076C>T	p.Leu3026Phe	0	C0	***Deleterious***	good	Het	Spectrin repeat	
	X	153418535	OPN1LW	missense	NM_020061.4:c.532A>G	p.Ile178Val	3	C0	Tolerated	good	Homo	GPCR, rhodopsin-like, 7TM	

Segregates	Chr	Position	Gene	Coding Effect	cDNA change	Protein change	BLOSUM62	AGVGD class	SIFTprediction	MAPPprediction	Zygosity	Protein Domain	Reference
**MA19: male, family history suggests RCD and recessive inheritance**													
	1	216270422	USH2A	splicing?	NM_206933.2:c.4758+3A>G	p.?	NA	NA	NA	NA	Het		
	4	15982163	PROM1	splicing?	NM_006017.2:c.2374-4dupG	p.?	NA	NA	NA	NA	Het		
NO	4	6303810	WFS1	missense	NM_006005.3:c.2288A>C	p.His763Pro	***-2***	***C15***	***Deleterious***	***bad***	Het		
NO	4	15513014	CC2D2A	in-frame	NM_001080522.2:c.685_687delGAA	p.Glu229del	NA	NA	NA	NA	Het		
A	4	15539735	CC2D2A	missense	NM_001080522.2:c.1978G>C	p.Val660Leu	1	C0	Tolerated	good	Het		
	4	123665061	BBS12	missense	NM_152618.2:c.2014G>A	p.Ala672Thr	0	***C55***	***Deleterious***	***bad***	Het		
	5	90149261	GPR98	missense	NM_032119.3:c.17365A>G	p.Lys5789Glu	1	C0	Tolerated	good	Het		
	6	135787297	AHI1	missense	NM_017651.4:c.404A>C	p.Gln135Pro	***-1***	C0	Tolerated	unknown	Het		
	8	87645092	CNGB3	missense	NM_019098.4:c.1208G>A	p.Arg403Gln	1	C0	***Deleterious***	***bad***	Het		
	9	120476570	TLR4	missense	NM_138554.3:c.2164A>G	p.Ile722Val	3	***C25***	***Deleterious***	***bad***	Het	Toll/interleukin-1 receptor homology domain	
A	10	55582584	PCDH15	frameshift	NM_001142763.1:c.4923delT	p.Glu1642Argfs*5	NA	NA	NA	NA	Het		
A	10	55721600	PCDH15	missense	NM_001142763.1:c.2936A>C	p.Tyr979Ser	***-2***	C0	***Deleterious***	good	Het	Cadherin	
	12	76740134	BBS10	missense	NM_024685.3:c.1631A>G	p.Asn544Ser	1	C0	Tolerated	good	Het		
A	X	31854856	DMD	missense	NM_004006.2:c.7179A>C	p.Lys2393Asn	0	C0	***Deleterious***	***bad***	Het	Dystrophin/utrophin	

Segregates	Chr	Position	Gene	Coding Effect	cDNA change	Protein change	BLOSUM62	AGVGD class	SIFTprediction	MAPPprediction	Zygosity	Protein Domain	Reference
**MA20: male, family history suggests RP and recessive inheritance**													
	2	110904416	NPHP1	splicing?	NM_000272.3:c.1438-4C>T	p.?	NA	NA	NA	NA	Het		
	2	96955677	SNRNP200	missense	NM_014014.4:c.2800A>G	p.Thr934Ala	0	C0	Tolerated	good	Het		
	5	90149261	GPR98	missense	NM_032119.3:c.17365A>G	p.Lys5789Glu	1	C0	Tolerated	good	Het		
	6	42689868	PRPH2	missense	NM_000322.4:c.205G>T	p.Val69Leu	1	C0	Tolerated	good	Het	Tetraspanin	
	6	135776946	AHI1	missense	NM_017651.4:c.1270A>G	p.Ile424Val	3	C0	Tolerated	good	Het		
	11	76891457	MYO7A	missense	NM_000260.3:c.2624C>G	p.Ala875Gly	0	C0	Tolerated	***bad***	Het		
A	12	88472996	CEP290	missense	NM_025114.3:c.5237G>A	p.Arg1746Gln	1	C0	Tolerated	good	Het		
A	12	88519039	CEP290	missense	NM_025114.3:c.1173G>T	p.Glu391Asp	2	C0	Tolerated	good	Het		
NO	15	31318408	TRPM1	missense	NM_002420.4:c.3497A>T	p.His1166Leu	***-3***	C0	Tolerated	good	Homo		
A	17	58233966	CA4	missense	NM_000717.3:c.158C>T	p.Pro53Leu	***-3***	***C65***	***Deleterious***	***bad***	Het	Alpha carbonic anhydrase	
	17	63221207	RGS9	missense	NM_003835.3:c.1495T>C	p.Ser499Pro	***-1***	C0	***Deleterious***	unknown	Het		
A	X	32459413	DMD	missense	NM_004006.2:c.3805C>A	p.His1269Asn	1	C0	Tolerated	good	Het	Spectrin/alpha-actinin	
A	X	38163895	RPGR	missense	NM_001034853.1:c.927G>T	p.Leu309Phe	0	C0	***Deleterious***	good	Het	Regulator of chromosome condensation, RCC1	

Candidate variants after alignment, variant calling and filtering are shown. The chromosome and position of the variants are depicted according to the human genome assembly, hg19. For the missense variants, pathogenic scores are shown in bold and italicised text. So for BLOSUM62, high pathogenicity=<0; AGVGD, high pathogenicity=C15 to C65; SIFT prediction, high pathogenicity=deleterious; MAPP prediction, high pathogenicity=bad. Homo = homozygous. Het = heterozygous. Whether the missense variant lies within a protein domain is also shown. NA = not annotated. Segregation of the variants where DNA from additional family members was available is also highlighted. A = artefact, R = real. The variants confirmed to be pathogenic are highlighted in bold and underlined.
